# CuAAC click chemistry for the enhanced detection of novel alkyne-based natural product toxins†
†Electronic supplementary information (ESI) available. See DOI: 10.1039/c8cc05113e


**DOI:** 10.1039/c8cc05113e

**Published:** 2018-10-12

**Authors:** Edward S. Hems, Ben A. Wagstaff, Gerhard Saalbach, Robert A. Field

**Affiliations:** a Department of Biological Chemistry, John Innes Centre , Norwich Research Park , Norwich , NR4 7UH , UK . Email: rob.field@jic.ac.uk

## Abstract

In the context of discovering and quantifying terminal alkyne-based natural products, here we report the combination of CuAAC click chemistry with LC-MS for the detection of polyether toxins (prymnesins) associated with harmful algal blooms. The added-value of the CuAAC-based approach is evident from our ability to detect novel prymnesin-like compounds in algal species with previously uncharacterised toxins.

## Abstract 2

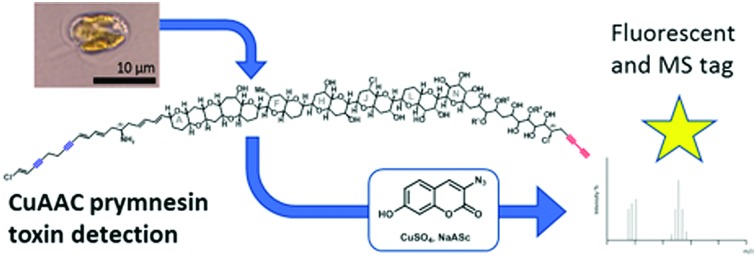
In the context of discovering and quantifying terminal alkyne-based natural products, here we report the combination of CuAAC click chemistry with LC-MS for the detection of polyether toxins (prymnesins) associated with harmful algal blooms.

## 

*Prymnesium parvum* is a toxin-producing microalga that causes harmful algal blooms globally, frequently leading to large-scale fish kills that damage ecosystems and the economy of the affected areas.^1^ We recently reported upon our efforts to tackle blooms of this microalga on the Norfolk Broads in the East of England.^2^–^4^ Although several toxic compounds have been reported from *P. parvum*,^5^–^7^ the toxins responsible for fish kills are believed to be the ichthyotoxic prymnesins-potent ladder-frame polyethers decorated with uncommon structural features, such as chlorine and alkynes (Fig. 1). First discovered and structurally characterized in 1996,^8^,^9^ the prymnesins were only relatively recently detected by other research groups,^10^,^11^ who have suggested that a challenge may lie in the chemodiversity of this class of toxins.^11^ Initially aiming to employ CuAAC derivatization of the terminal alkyne group present in prymnesins for their facile fluorescence detection, herein we illustrate how this chemistry is invaluable for enhancing LC-MS-based detection of new alkyne-based natural product toxins.

**Fig. 1:**
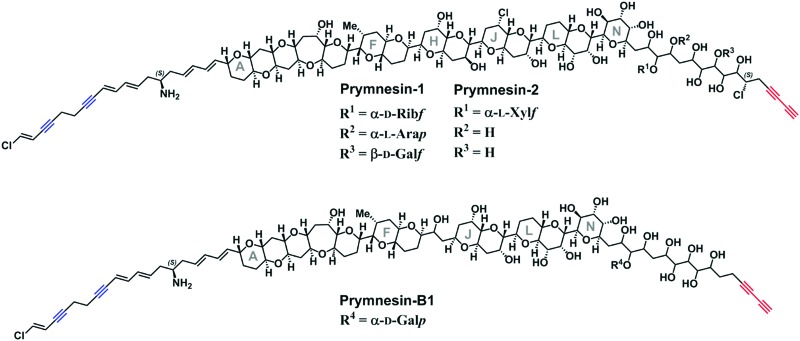
The reported structures of the known prymnesin toxins.^8^,^9^,^11^ Blue = internal alkynes. Red = terminal bis-alkyne. Note that despite the variation between prymnesin-1/2 and prymnesin-B1 toxins, the number and relative locations of the alkynes are conserved.

Current classification of the prymnesins groups them into either A-, B- or C-type based on their polyether core, sub-divided by the extent of chlorination and the nature and extent of their glycosylation (Fig. 1).^11^ Detection of prymnesins typically relies upon LC-MS analysis^10^,^11^ or labelling of the conserved amine group with ninhydrin and phenylacetaldehyde to give a fluorescent adduct.^12^ We were drawn to consider whether the conserved terminal bis-alkyne on prymnesin toxins might prove to be a useful chemical handle for the detection of prymnesins *via* the ubiquitous bioorthogonal copper-catalysed alkyne-azide cycloaddition (CuAAC) ‘click’ reaction, which has been deployed in a number of diagnostic contexts.^13^ The detection of a terminal alkyne-based plant natural product using this approach was reported recently.^14^ Terminal bis-alkynes, as in the prymnesins, are rare moieties in nature, with interesting chemical and spectroscopic properties.^15^,^16^ We were therefore minded to explore the possibility of specifically labelling prymnesins through CuAAC click chemistry to aid their detection and characterisation. Since it is challenging to obtain quantities of prymnesins from laboratory scale cultures,^8^,^11^ preliminary experiments required synthetic terminal alkyne (**1**) and bis-alkyne (**2**), which were prepared as set out in Scheme S1 (ESI†). In trial reactions with 3-azido-7 hydroxycoumarin (**3**),^17^,^18^ CuAAC reaction efficiencies for alkyne **1** and bis-alkyne **2** were broadly similar (Fig. S1, ESI†), showing that terminal bis-alkynes are indeed good candidates for targeted CuAAC click chemistry (Fig. 2).

**Fig. 2:**
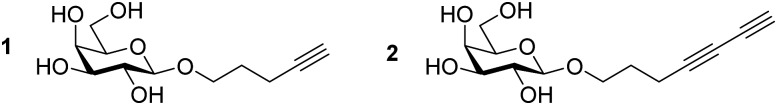
Target mono- (**1**) and bis-alkyne (**2**) prymnesin model compounds produced synthetically as set out in Scheme S1 (ESI†).

Next, we investigated whether CuAAC click chemistry could be utilised to detect prymnesin toxins from laboratory cultures of *Prymnesium* (Table S1, ESI†). Previous work^10^,^11^ has shown that prymnesins display diagnostic isotope patterns in MS data, in part related to the number of chlorine atoms present. Furthermore, prymnesins fly as double-charged ions, fragmenting readily to the aglycone form of the toxin, which is often the most abundant ion present. We first sought to use CuAAC click chemistry to detect prymnesins from *P. parvum* (946/6), a strain which is known to produce the ‘A-type’ prymnesins-1 and -2 (referred to as prymnesin-A1 and -A2 hereafter).^19^ Prymnesin toxins were extracted from lab grown cultures of *Prymnesium* using a modification of published procedures^10^ and subjected to LC-MS analysis. Using this approach, we could identify signals for previously characterized prymnesin-A1 and -A2 from *P. parvum* 946/6 (Fig. 3 and Fig. S3, ESI†), which we also later detected in the *P. parvum* 94A strain (Fig. S4, ESI†). The extracts containing the ‘A-type’ prymnesins were subjected to CuAAC click chemistry with **3** under similar conditions to those described for model alkynes **1** and **2**. If the click reaction was successful, we would expect the formation of new prymnesin-like molecules coupled to the coumarin chromophore *via* a triazole ring. Before re-examining these clicked extracts using LC-MS, they were subjected to TLC analysis,^10^ which confirmed the formation of new products visible by long wave UV (365 nm) (Fig. S2, ESI†). In future, this TLC analysis may be exploited as a tool to simply assess the presence of toxins of this type in other algal strains or even natural water samples.

**Fig. 3:**
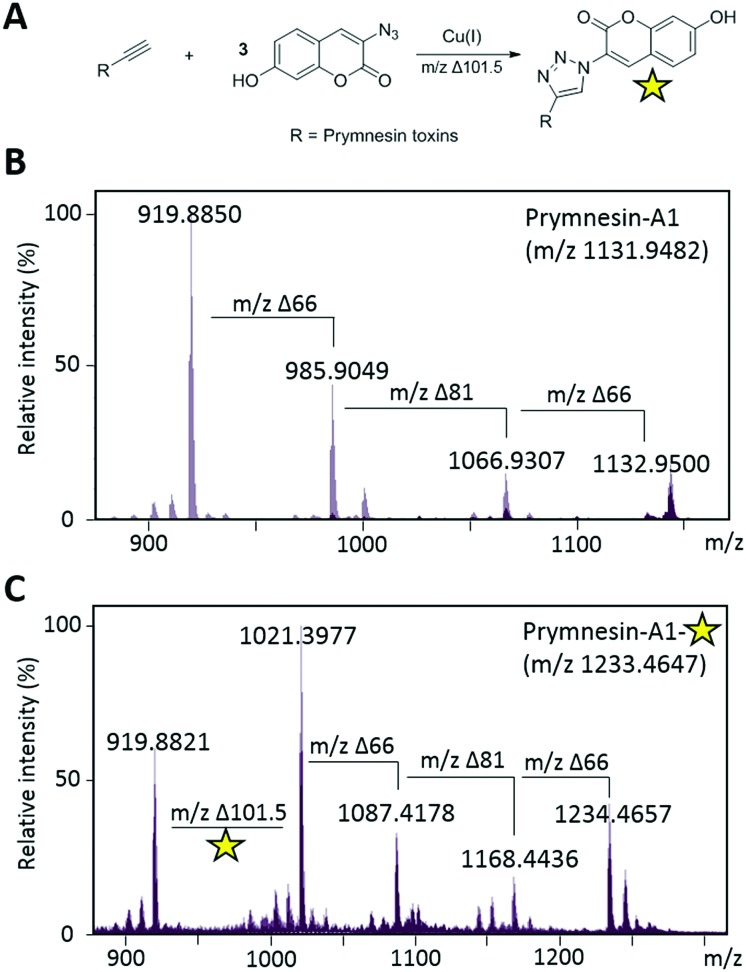
Click coupling of prymnesin-A1 from *P. parvum* 946/6 culture extracts. (A) Reaction scheme for the CuAAC click coupling of 3-azido-7-hydroxycoumarin (**3**) with prymnesin toxins. Yellow star represents the activated fluorescent triazole product. (B) MS/HRMS spectra of [M + 2H]^2+^ for previously reported prymnesin-A1. Δ66 reflects a loss of a pentose unit and Δ81 reflects a loss of a hexose unit. Prymnesin-A1 is triply glycosylated with 2× pentose units and 1× hexose. (C) MS/HRMS spectra of [M + 2H]^2+^ for prymnesin-A1 after CuAAC click coupling with **3**. Δ101.5 reflects addition of the coumarin moiety to the prymnesin toxin. Fragmentation of pentose and hexose units is still visible after addition of **3**.

Next, the clicked extracts were examined by LC-MS to see if masses corresponding to the ‘clicked’ prymnesin-A1 and -A2 toxins could be identified. Signals previously assigned as originating from these toxins were found to increase in mass by the expected value of *m*/2 ∼101.5 for the addition of 3-azido-7-hydroxycoumarin (**3**) (Fig. 3A). For prymnesin-A1 and -A2, which share the same backbone mass (aglycone) of ∼918.9 (*m*/2 ion), this click conjugation with **3** was reflected by an increase in mass of ∼101.5 to ∼1020.4 (Fig. 3C). The reaction with **3** did not affect the fragmentation pattern of the toxins, with prymnesin-A1 showing *m*/2 losses of 66 and 81, corresponding to losses of 2 pentose units and 1 hexose from the intact form of the toxin (Fig. 3C). Prymnesin-A2 is only glycosylated at a single position with a pentose, and subsequently shows only 1 fragmentation loss of *m*/2 66 corresponding to the loss of this pentose. Measured isotope distribution patterns for both the non-clicked and clicked toxins match theoretical predictions calculated using enviPat^20^ (Fig. S7 and S8, ESI†).

Using this methodology, we next identified prymnesin-like compounds from the *P.* sp. 595 strain with a similar backbone composition to those of the previously reported ‘B-type’ toxins,^11^ which we have termed prymnesin-B6 and -B7 (Fig. S5, ESI†). We also detected signals from this strain for prymnesin-B1 and the non-glycosylated form of prymnesin-B1, but at much lower intensities (Table S2, ESI†). Prymnesin-B6 and -B7 show increased levels of chlorination compared to prymnesin-B and -B1,^11^ which is reflected by the corresponding double charged ions of the backbone of prymnesin-B6 and -B7 being *m*/2 17 units higher. Prymnesin-B6 shows no obvious fragmentation, and we therefore speculate this compound is not glycosylated. Prymnesin-B7 shares the same backbone as B6 but shows a single fragmentation loss of *m*/*z* 81, corresponding to the loss of a hexose. Upon CuAAC click coupling of these putative toxins with azidocoumarin **3**, both show increases in their backbone masses from ∼845.9 by ∼101.5 units to ∼947.4, suggesting that these compounds also share the terminal alkyne system observed in all prymnesins reported to date. As with prymnesin-A1 and -A2, click coupling to **3** did not alter fragmentation patterns and measured isotope distribution patterns match theory (Fig. S9, ESI†). Due to the much lower intensities of prymnesin-B and -B1 in the extracts, signals corresponding to the clicked products of these toxins could not be observed.

We could find no evidence for the previously reported ‘C-type’ prymnesins^11^ in any of the 4 strains that we examined. However, in *P. patelliferum* 527D, we detected signals for novel alkyne-containing molecules with distinct backbone masses and elemental compositions. The nature of the toxin produced by this *Prymnesium* genus has yet to be confirmed.^21^ We termed these compounds prymnesin-D1 to -D4, and suggest that they make up a new ‘D-type’ family of prymnesin toxins, based on positive CuAAC derivatization (Fig. 4). Prymnesin-D1 and -D2 share a *m*/2 backbone mass of ∼883.8 and are glycosylated in a similar manner to prymnesin-A1 and -A2: D1 shows fragmentation loss of 2 pentose units and 1 hexose from the intact compound (Fig. 4), whilst -D2 shows a single fragmentation loss of 1 pentose. Prymnesin-D3 and -D4, respectively, share these glycosylation patterns, but have backbone masses *m*/2 18 units lower, corresponding to the absence of HCl (Table 1). This lack of HCl suggests the presence of an additional alkene or alkyne moiety, with loss of chlorine, in the toxin backbone. As we do not observe multiple additions of **3** to these toxins, we speculate that the absence of HCl results in a new internal alkene at the glycosylated ‘tail’ of the toxins or in the polyether ring system (Fig. 1). These D-type toxins display similar levels of unsaturation to the A-type prymnesins, suggesting more alkenes or conjugated ring systems are present in these toxins than in the B and C-type (Table S1, ESI†).

**Fig. 4:**
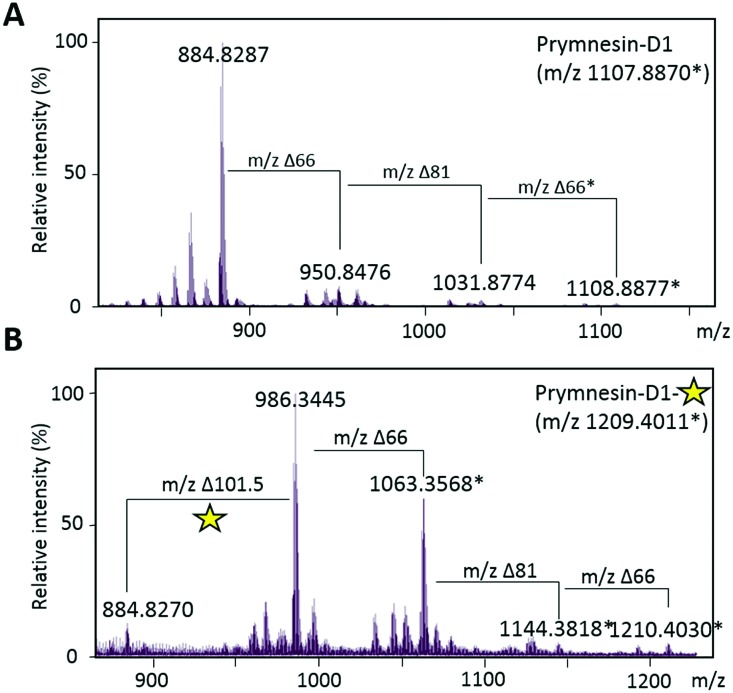
Click coupling of putative novel prymnesin-D1 from *P. patelliferum* 527D culture extracts. (A) MS/HRMS spectra of [M + 2H]^2+^ for prymnesin-D1. Δ66 reflects a loss of a pentose unit and Δ81 reflects a loss of a hexose unit. Prymnesin-D1 is triply glycosylated with 2× pentose units and 1× hexose. ‘*’ represents the [M + Na + H]^2+^ ion. (B) MS/HRMS spectra of [M + 2H]^2+^ for prymnesin-D1 after CuAAC click coupling with **3**. Δ101.5 reflects addition of the coumarin moiety to the prymnesin toxin. Fragmentation of pentose and hexose units is still visible after addition of **3**. Yellow star represents the activated fluorescent triazole product. ‘*’ represents the [M + Na + H]^2+^ ion.

**Table 1:** *Prymnesium* strains and corresponding HRMS identification of 8 prymnesin compounds, including prymnesin-A1 and -A2 originally reported by Igarashi *et al.*,^8^,^9^ based on MS/HRMS and subsequent labelling with 3-azido-7-hydroxycoumarin (**3**). Masses reported correspond to [M + 2H]^2+^ ion, unless denoted with a ‘*’ which reports the [M + Na + H]^2+^ ion. Toxins that share the same backbone only have 1 value reported for the clicked aglycone, which is an average signal for the aglycone from all toxins

Strain	Prymnesin-type	Elemental composition of aglycone	[M-glycone + 2H]^2+^	Elemental composition	[M + 2H]^2+^ [M + Na + H]^2+^*	Elemental composition of ‘clicked’ toxin (aglycone)	[M-glycone + azidocoumarin + 2H]^2+^
*P. parvum* 946/6	Prymnesin-Al	C_91_H_128_CI_3_NO_31_ (Δ0.6 ppm)	918.8835	C_107_H_154_CUNO_44_ (Δ4.0 ppm)	1131.9482	C_100_H_133_CUN_4_O_34_ (Δ4.0 ppm)	1020.3965
	Prymnesin-A2	C_91_H_128_CI_3_NO_31_ (Δ1.3 ppm)	918.8853	C_96_H_136_Cl_3_NO_35_ (Δ1.5 ppm)	984.9037		

*P. parvum* 94A	Prymnesin-Al	Cg_1_H_128_Cl_3_NO_31_ (Δ4.6 ppm)	918.8798	C_107_H_15_CUNO_44_ (Δ6.0 ppm)	1131.9460	C_100_H_133_Cl_3_N_4_O_34_ (Δ6.4 ppm)	1020.3941
	Prymnesin-A2	Cg_1_H_128_Cl_3_NO_31_ (Δ4.2 ppm)	918.8802	C_96_H_136_Cl_3_NO_35_ (Δ5.5 ppm)	984.8998		

*P.* sp. 595	Prymnesin-B6	C_85_H_121_Cl_2_NO_29_ (Δ2.1 ppm)	845.8756	C_85_H_121_Cl_2_NO_29_ (Δ2.1 ppm)	845.8756	C_94_H_126_Cl_2_N_4_O_32_ (Δ5.8 ppm)	947.3884
	Prymnesin-B7	C_85_HmCl_2_NO_29_ (Δ3.4 ppm)	845.8745	C_91_H_131_Cl_2_NO_34_ (Δ4.9 ppm)	926.8992		

*P. patelliferum* 527D	Prymnesin-Dl	C_85_H_114_Cl_3_NO_32_ (Δ0.3 ppm)	883.8270	C_101_H_140_CUNO_45_ (Δ0.5 ppm)	1107.8870*	C_94_H_119_Cl_3_N_4_O_35_ (Δ0.4 ppm)	985.3437
	Prymnesin-D2	C_85_H_114_Cl_3_NO_32_ (Δ3.4 ppm)	883.8298	C_90_H_122_Cl_3_NO_36_ (Δ0.5 ppm)	949.8484		
	Prymnesin-D3	C_85_H_113_Cl_2_NO_32_ (Δ0.2 ppm)	865.8386	C_101_H_139_ChNO_45_ (Δ0.7 ppm)	1078.9063	C_94_H_118_G_2_N_4_O_35_ (Δ4.1 ppm)	967.3510
	Prymnesin-D4	C_85_H_113_Cl_2_NO_32_ (Δ0.4 ppm)	865.8388	C_90_H_121_Cl_2_NO_36_ (Δ0.7 ppm)	931.8589		

Successful CuAAC ‘click’ coupling of these novel D-type prymnesin-like-compounds confirmed the presence of a single terminal alkyne system, as we also observe mass shifts corresponding to the addition of **3** to these compounds. For prymnesin-D1 and -D2, the double charged backbone mass of ∼883.8 increases by ∼101.5 units to ∼985.3 (Fig. 4B), whilst for prymnesin-D3 and -D4, the double charged backbone mass of ∼865.8 increases by ∼101.5 units to ∼967.3 (Table S2 and Fig. S6, ESI†). In each case, fragmentation patterns still show the loss of monosaccharides from the intact compounds, while measured isotope patterns match theory well (Fig. S10, ESI†). A summary of the major prymnesins found in this study, along with their corresponding masses, can be seen in Table 1.

The discovery of prymnesin-A1 and -A2 from 2 out of 4 strains examined extends the findings of Rasmussen *et al.*,^11^ who only found these particular toxins in 1/10 strains that they examined (*i.e.* UTEX-2797), suggesting that these toxins are more widespread than previously thought. In addition, the C-type prymnesins were found in 5/10 strains examined by Rasmussen *et al.*,^11^ where we did not find them in any of the 4 strains in this study. Instead, the discovery of a new type of prymnesin-like toxin from *P. patelliferum* 527D, the D-type, shows that more species and strains of *Prymnesium* need to be studied to obtain a more complete picture of the global abundance and chemodiversity of these toxins. Interestingly, Rasmussen *et al.*^11^ hinted at a regionality to the toxin types (*i.e.* they found mostly B-type from strains originally isolated from Scandinavia). Our results broadly agree with this suggestion, with an A-type producer originally isolated from North America (*P. parvum* 94A), and the B-type producer originally isolated off the coast of Finland (*P*. sp. 595). A summary of the strains used in this study, their place of isolation, and the type of prymnesins that they produce, along with the previous findings of Rasmussen *et al.*,^11^ can be found in ESI,† Table S1.

Overall, the current study adds a putative new type of prymnesins to the A, B and C-types previously reported.^8^–^11^ Being only the second study to report on novel prymnesin structures, we expect that many more variants are yet to be discovered, as has been seen for the growing number of algal polyether toxins over the last 2 decades.^22^ This study also shows the added value of CuAAC click derivatization, in parallel with LCMS analysis, in aiding the detection and discovery of terminal alkyne-containing natural products that are otherwise difficult to identify.

## Conflicts of interest

There are no conflicts to declare.

## Supplementary Material

Supplementary information

Supplementary information
