# Correction: CuAAC click chemistry for the enhanced detection of novel alkyne-based natural product toxins

**DOI:** 10.1039/c8cc90504e

**Published:** 2018-11-13

**Authors:** Edward S. Hems, Ben A. Wagstaff, Gerhard Saalbach, Robert A. Field

**Affiliations:** Department of Biological Chemistry, John Innes Centre Norwich Research Park Norwich NR4 7UH UK rob.field@jic.ac.uk

## Abstract

Correction for 'CuAAC click chemistry for the enhanced detection of novel alkyne-based natural product toxins' by Edward S. Hems *et al.*, *Chem. Commun.*, 2018, **54**, 12234–12237.

The authors regret that Table 1 was displayed incorrectly in the original article. The correct version is shown below.

**Table 1:** Prymnesium strains and corresponding HRMS identification of 8 prymnesin compounds, including prymnesin-A1 and -A2 originally reported by Igarashi *et al.*,^8,9^ based on MS/HRMS and subsequent labelling with 3-azido-7-hydroxycoumarin (**3**). Masses reported correspond to [M + 2H]^2+^ ion, unless denoted with a ‘*’ which reports the [M + Na + H]^2+^ ion. Toxins that share the same backbone only have 1 value reported for the clicked aglycone, which is an average signal for the aglycone from all toxins

Strain	Prymnesin-type	Elemental composition of aglycone	[M-glycone + 2H]^2+^	Elemental composition	[M + 2H]^2+^ [M + Na + H]^2+^*	Elemental composition of ‘clicked’ toxin (aglycone)	[M-glycone + azidocoumarin + 2H]^2+^
*P. parvum* 946/6	Prymnesin-A1	C_91_H_128_Cl_3_NO_31_ (Δ0.6 ppm)	918.8835	C_107_H_154_Cl_3_NO_44_ (Δ4.0 ppm)	1131.9482	C_100_H_133_Cl_3_N_4_O_34_ (Δ4.0 ppm)	1020.3965
	Prymnesin-A2	C_91_H_128_Cl_3_NO_31_ (Δ1.3 ppm)	918.8853	C_96_H_136_Cl_3_NO_35_ (Δ1.5 ppm)	984.9037	"	"

*P. parvum* 94A	Prymnesin-A1	C_91_H_128_Cl_3_NO_31_ (Δ4.6 ppm)	918.8798	C_107_H_154_Cl_3_NO_44_ (Δ6.0 ppm)	1131.9460	C_100_H_133_Cl_3_N_4_O_34_ (Δ6.4 ppm)	1020.3941
	Prymnesin-A2	C_91_H_128_Cl_3_NO_31_ (Δ4.2 ppm)	918.8802	C_96_H_136_Cl_3_NO_35_ (Δ5.5 ppm)	984.8998	"	"

*P.* sp. 595	Prymnesin-B6	C_85_H_121_Cl_2_NO_29_ (Δ2.1 ppm)	845.8756	C_85_H_121_Cl_2_NO_29_ (Δ2.1 ppm)	845.8756	C_94_H_126_Cl_2_N_4_O_32_ (Δ5.8 ppm)	947.3884
	Prymnesin-B7	C_85_H_121_Cl_2_NO_29_ (Δ3.4 ppm)	845.8745	C_91_H_131_Cl_2_NO_34_ (Δ4.9 ppm)	926.8992	"	"

*P. patelliferum* 527D	Prymnesin-D1	C_85_H_114_Cl_3_NO_32_ (Δ0.3 ppm)	883.8270	C_101_H_140_Cl_3_NO_45_ (Δ0.5 ppm)	1107.8870*	C_94_H_119_Cl_3_N_4_O_35_ (Δ0.4 ppm)	985.3437
	Prymnesin-D2	C_85_H_114_Cl_3_NO_32_ (Δ3.4 ppm)	883.8298	C_90_H_122_Cl_3_NO_36_ (Δ0.5 ppm)	949.8484	"	"
	Prymnesin-D3	C_85_H_113_Cl_2_NO_32_ (Δ0.2 ppm)	865.8386	C_101_H_139_Cl_2_NO_45_ (Δ0.7 ppm)	1078.9063	C_94_H_118_Cl_2_N_4_O_35_ (Δ4.1 ppm)	967.3510
	Prymnesin-D4	C_85_H_113_Cl_2_NO_32_ (Δ0.4 ppm)	865.8388	C_90_H_121_Cl_2_NO_36_ (Δ0.7 ppm)	931.8589	"	"

The Royal Society of Chemistry apologises for these errors and any consequent inconvenience to authors and readers.

## Supplementary Material

