# Supplementary material for: CuAAC click chemistry for the enhanced detection of novel alkyne-based natural product toxins
Source: Chem Commun (Camb). 2018 Oct 12;54(86):12234–7. doi: 10.1039/c8cc05113e (PMC6243676; doi:10.1039/c8cc05113e)
Supplement: Supplementary file 1 [file CC-054-C8CC05113E-s001.pdf]

Electronic Supplementary Material (ESI) for Chemical Communications.
This journal is © The Royal Society of Chemistry 2018

# Supplementary Information

# CuAAC click chemistry for the enhanced detection of novel alkyne-based natural product toxins

**Edward S. Hems,[‡a] Ben A. Wagstaff,[‡a] Gerhard Saalbach[a] and Robert A. Field*[a]**

## Contents

**Scheme S1** .......... 3

**Table S1** .......... 4

**Figure S1** .......... 5

**Figure S2** .......... 6

**Figure S3** .......... 6

**Figure S4** .......... 7

**Figure S5** .......... 8

**Figure S6** .......... 9

**Figure S7** .......... 10

**Figure S8** .......... 10

**Figure S9** .......... 11

**Figure S10** .......... 12

General experimental conditions .......... 13

Extraction of prymnesin toxins from *P. parvum* cell cultures .......... 14

CuAAC coupling of prymnesin extracts .......... 14

LC-MS detection of prymnesin toxins .......... 14

Chemistry .......... 15

(Bromoethynyl) triethylsilane[4] .......... 15

Pent-4-yn-1-yl 2′,3′,4′,6′-tetra-O-acetyl-β-ᴅ-galactopyranoside (5)[5] .......... 15

Pent-4-yn-1-yl β-ᴅ-galactoside (1)[5] .......... 16

7-(Triethylsilyl)hepta-4,6-diyn-yl-β-D-galactoside (6) .......... 16

Hepta-4,6-diyn-yl-β-D-galactoside (2) .......... 17

3-azido-7-hydroxycoumarin (3)[7] .......... 17

3-[1-(7-Hydroxy-coumarin)-1H-1,2,3-triazol-4-yl] propyl β-D-galactopyranoside (7) .......... 18

5-[1-(7-Hydroxy-coumarin)-1H-1,2,3-triazol-4-yl] pent-4-yn-1-yl β-D-galactopyranoside (8) .......... 19

NMR Spectra: .......... 20

(Bromoethynyl) triethylsilane .......... 20

Pent-4-yn-1-yl 2',3',4',6'-tetra-O-acetyl-β-D-galactopyranoside (5) .......... 21

Pent-4-yn-1-yl β-D-galactoside (1) .......... 22

7-(Triethylsilyl)hepta-4,6-diyn-yl-β-D-galactoside (6) .......... 23

Hepta-4,6-diyn-yl-β-D-galactoside (2) .......... 24

3-azido-7-hydroxycoumarin (3) .......... 25

3-[1-(7-Hydroxy-coumarin)-1H-1,2,3-triazol-4-yl] propyl β-D-galactopyranoside (7) .......... 26

5-[1-(7-Hydroxy-coumarin)-1H-1,2,3-triazol-4-yl] pent-4-yn-1-yl β-D-galactopyranoside (8) .......... 28

References: .......... 29

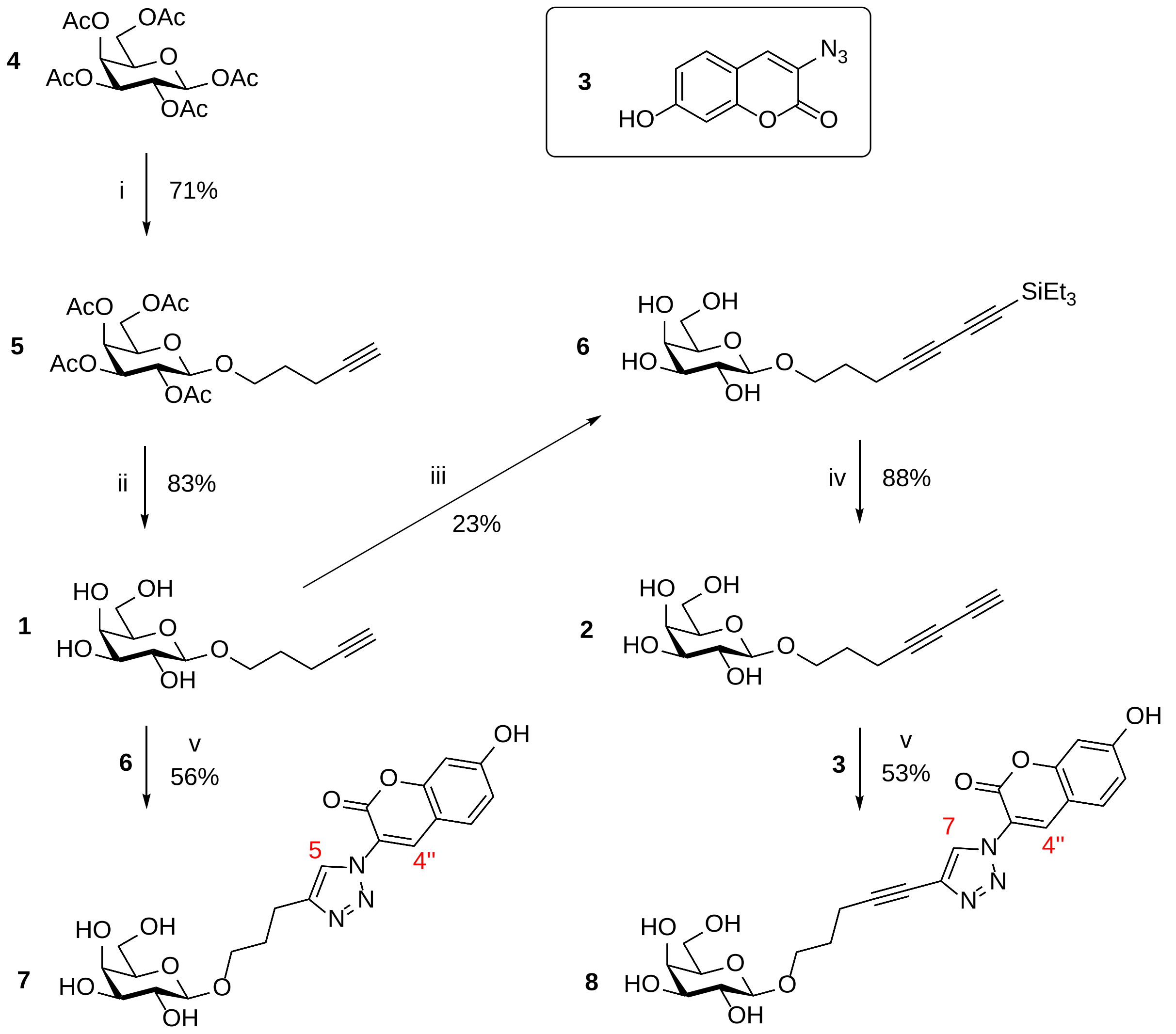


**Scheme S1** - Synthesis of mono- (**1**) and bis-alkyne (**2**) prymnesin model compounds and the model click reactions to produce **7** and **8**. i) 4-pentyn-1-ol, $BF_3.Et_2O$. ii) Na, MeOH. iii) Br-C≡C-TES, Cu(I)Cl, $BuNH_2$. MeOH. iv) $NEt_3.3HF$. v) MeOH:$H_2O$ (1:1), $CuSO_4$, NaAsc.

| | Name | Elemental Composition | $[M+2H]^{2+}$ (Measured) | Backbone $[M+2H]^{2+}$ (Measured) | Elemental Composition of backbone | DBE | Strain: *P. parvum* (UTEX-2797) | *P. parvum* (CCAP 946/6) | *P. parvum* (94A) | *P. parvum* (K-0081) | *P. parvum* (K-0374) | *P. parvum* (KAC-39) | *P.* sp. (595) | *P. parvum* (RCC-1433) | *P. parvum* (RCC-1436) | *P. parvum* (RCC-191) | *P. parvum* (RCC-1435) | *P. parvum* (N-1017) | *P. parvum* (K-0252) | *P. patelliferum* (527D) |
|---|---|---|---|---|---|---|---|---|---|---|---|---|---|---|---|---|---|---|---|---|
| **Collection location** | | | | | | | Texas, USA | Isle of Cumbrae, Scotland | English Bay, Br. Columbia, Canada | Flade lake, Denmark | Oslo Fjord, Norway | Norway | Baltic Sea, Finland | English Channel, France | Atlantic Ocean, French Coast | Dorset, England | La Reunion, Indian Ocean | Japan | Australia | North of Buesum, Germany, North Sea |
| **A-type** | Prymnesin-1 | $C_{107}H_{154}Cl_3NO_{44}$ (Δ 4.0 ppm) | 1131.9482 | 918.8835 | $C_{91}H_{128}Cl_3NO_{31}$ (Δ 0.6 ppm) | 30 | + | + | + | | | | | | | | | | | |
| | Prymnesin-2 | $C_{96}H_{136}Cl_3NO_{35}$ (Δ 1.5 ppm) | 984.9037 | 918.8853 | $C_{91}H_{128}Cl_3NO_{31}$ (Δ 1.3 ppm) | 28 | + | + | + | | | | | | | | | | | |
| | | $C_{96}H_{137}Cl_2NO_{35}$ (Δ 0.01 ppm) | 967.9247 | 901.9037 | $C_{91}H_{129}Cl_2NO_{31}$ (Δ 0.2 ppm) | 28 | + | | | | | | | | | | | | | |
| **B-type** | Prymnesin-B1 | $C_{91}H_{132}ClNO_{34}$ (Δ 0.04 ppm) | 909.9232 | 828.8965 | $C_{85}H_{122}ClNO_{29}$ (Δ 0.4 ppm) | 26 | | | | + | | | | | | | | | + | |
| | Prymnesin-B2 | $C_{90}H_{130}ClNO_{33}$ (Δ 0.05 ppm) | 894.9180 | 828.8960 | $C_{85}H_{122}ClNO_{29}$ (Δ 1.0 ppm) | 26 | | | | + | + | + | | | | | | | + | |
| | | $C_{96}H_{140}ClNO_{38}$ (Δ 0.07 ppm) | 975.9443 | 828.8974 | $C_{85}H_{122}ClNO_{29}$ (Δ 0.7 ppm) | 27 | | | | + | + | + | | | | | | | + | |
| | | $C_{97}H_{142}ClNO_{39}$ (Δ 1.7 ppm) | 990.9480 | 828.8962 | $C_{85}H_{122}ClNO_{29}$ (Δ 0.8 ppm) | 27 | | | | + | | | | | | | | | | |
| | | $C_{85}H_{122}ClNO_{29}$ (Δ 0.03 ppm) | 828.8968 | 828.8968 | $C_{85}H_{122}ClNO_{29}$ (Δ 0.03 ppm) | 25 | | | | + | + | + | | | | | | | + | |
| | Prymnesin-B6 | $C_{85}H_{121}Cl_2NO_{29}$ (Δ 2.0 ppm) | 845.8756 | 845.8756 | $C_{85}H_{121}Cl_2NO_{29}$ (Δ 2.0 ppm) | 24 | | | | | | | + | | | | | | | |
| | Prymnesin-B7 | $C_{91}H_{131}Cl_2NO_{34}$ (Δ 4.9 ppm) | 926.8992 | 845.8745 | $C_{85}H_{121}Cl_2NO_{29}$ (Δ 3.4 ppm) | 25 | | | | | | | + | | | | | | | |
| **C-type** | | $C_{88}H_{125}Cl_2NO_{35}$ (Δ 0.04 ppm) | 913.8777 | 847.8549 | $C_{83}H_{117}Cl_2NO_{31}$ (Δ 2.0 ppm) | 26 | | | | | | | | + | + | + | + | + | | |
| | | $C_{88}H_{127}Cl_2NO_{35}$ (Δ 0.04 ppm) | 914.8856 | 848.8630 | $C_{83}H_{119}Cl_2NO_{31}$ (Δ 1.7 ppm) | 25 | | | | | | | | | + | + | | + | | |
| | | $C_{93}H_{135}Cl_2NO_{39}$ (Δ 3.0 ppm) | 980.9037 | 848.8642 | $C_{83}H_{119}Cl_2NO_{31}$ (Δ 0.3 ppm) | 26 | | | | | | | | | + | | | | | |
| | | $C_{88}H_{126}Cl_3NO_{35}$ (Δ 0.02 ppm) | 931.8661 | 865.8431 | $C_{83}H_{118}Cl_3NO_{31}$ (Δ 2.1 ppm) | 25 | | | | | | | | + | + | + | + | + | | |
| | | $C_{88}H_{124}Cl_3NO_{35}$ (Δ 2.7 ppm) | 930.8557 | 864.8457 | $C_{83}H_{116}Cl_3NO_{31}$ (Δ 1.6 ppm) | 26 | | | | | | | | + | + | + | | + | | |
| | | $C_{99}H_{144}Cl_3NO_{44}$ (Δ 1.2 ppm) | 1078.9123 | 865.8461 | $C_{83}H_{118}Cl_3NO_{31}$ (Δ 1.3 ppm) | 27 | | | | | | | | + | + | + | | | | |
| | | $C_{93}H_{134}Cl_3NO_{39}$ (Δ 2.1 ppm) | 997.8851 | 865.8437 | $C_{83}H_{118}Cl_3NO_{31}$ (Δ 1.4 ppm) | 26 | | | | | | | | + | + | | + | + | | |
| | | $C_{88}H_{125}Cl_4NO_{35}$ (Δ 0.5 ppm) | 948.8461 | 882.8254 | $C_{83}H_{117}Cl_4NO_{31}$ (Δ 0.1 ppm) | 25 | | | | | | | | + | + | + | + | + | | |
| **D-type** | Prymnesin-D1 | $C_{101}H_{140}Cl_3NO_{45}$ (Δ 7.5 ppm) | 1096.8872 | 883.827 | $C_{85}H_{114}Cl_3NO_{32}$ (Δ 0.3 ppm) | 30 | | | | | | | | | | | | | | + |
| | Prymnesin-D2 | $C_{90}H_{122}Cl_3NO_{36}$ (Δ 0.5 ppm) | 949.8484 | 883.8298 | $C_{85}H_{114}Cl_3NO_{32}$ (Δ 3.4 ppm) | 28 | | | | | | | | | | | | | | + |
| | Prymnesin-D3 | $C_{101}H_{139}Cl_2NO_{45}$ (Δ 0.7 ppm) | 1078.9063 | 865.8386 | $C_{85}H_{113}Cl_2NO_{32}$ (Δ 0.2 ppm) | 31 | | | | | | | | | | | | | | + |
| | Prymnesin-D4 | $C_{90}H_{121}Cl_2NO_{36}$ | 931.8589 | 865.8388 | $C_{85}H_{113}Cl_2NO_{32}$ | 29 | | | | | | | | | | | | | | + |

**Table S1** – *P. parvum* strains and their place of isolation as well as HR-MS tentative identification of prymnesin compounds. Compounds identified in red are newly identified compounds in this study. Table reproduced and adapted with permission from S. A. Rasmussen *et al., J. Nat. Prod.,* 2016, **79**, 2250-2256 (https://pubs.acs.org/doi/abs/10.1021%2Facs.jnatprod.6b00345). Further permissions related to the data in this table from the aforementioned publication should be directed to the ACS.

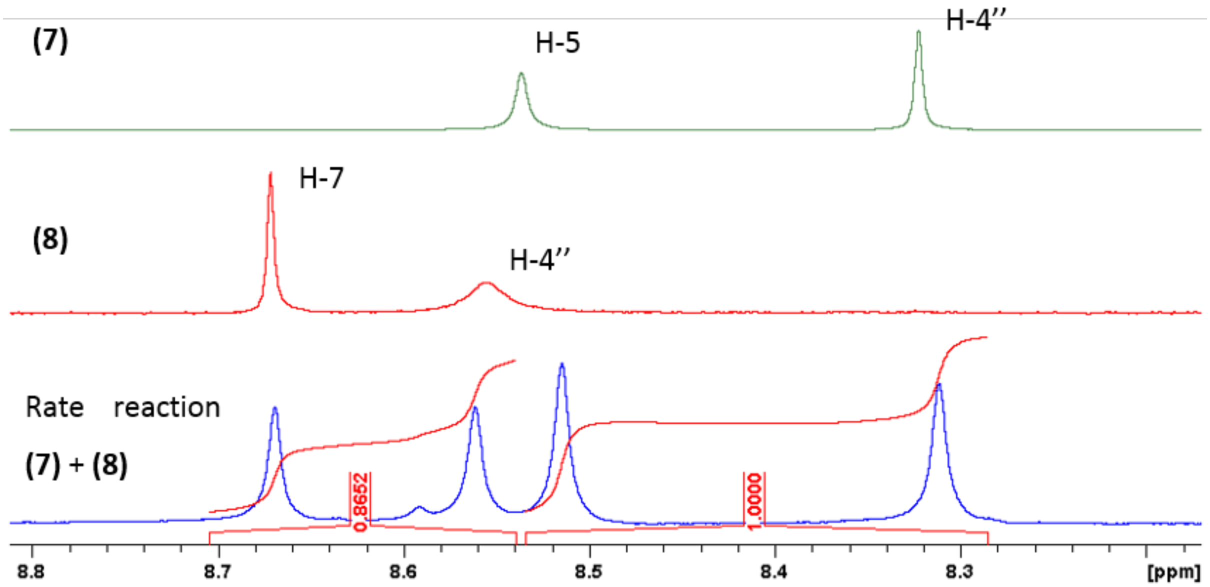


**Figure S1** - $^{1}$H NMR spectra showing the H-5 and H-4'' signals of **7** (green), H-7 and H-4'' signals of **8** (red) and a mixture of both compounds from the CuAAC reaction (blue). The ratios of **7:**8 were found to be 1:0.87.

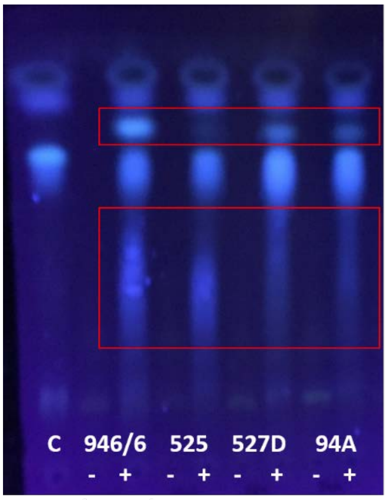


**Figure S2** – Prymnesium extracts from 4 strains reacted with 3-azido-7-hydroxycoumarin visualized by long wave UV (365 nm). C = control reaction of 3-azido-7-hydroxycoumarin with copper sulfate and sodium ascorbate in 50% EtOH. '-' = extract without addition of CuAAC reagents. '+' = extract with CuAAC reagents. Formation of new spots visible by long wave UV are highlighted in red boxes.

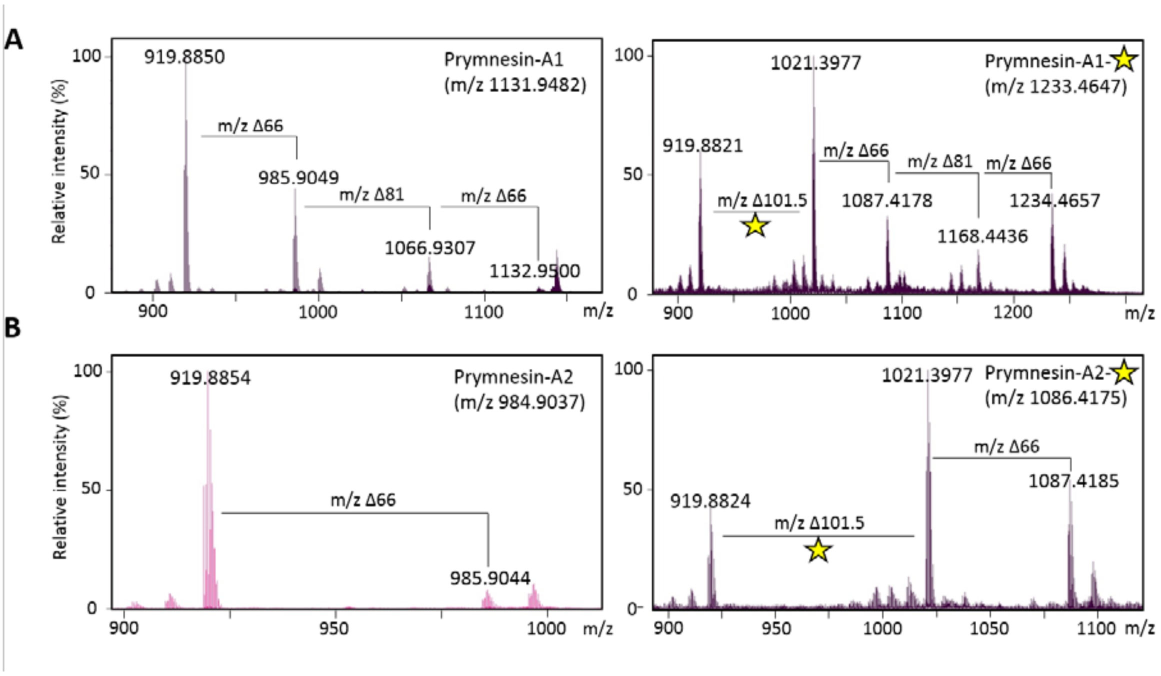


**Figure S3**– CuAAC click coupling of Prymnesin-A1 and -A2 from *P. parvum* 946/6 with 3-azido-7-hydroxycoumarin (**3**). (**A**) Left - MS/HRMS spectra of $[M+2H]^{2+}$ for prymnesin-A1. Δ66 reflects a loss of a pentose unit and Δ81 reflects a loss of a hexose unit. Prymnesin-A1 is triply glycosylated with 2x pentose units and 1x hexose. Right - MS/HRMS spectra of $[M+2H]^{2+}$ for prymnesin-A1 after CuAAC click coupling with **3**. (**B**) Left - MS/HRMS spectra of $[M+2H]^{2+}$ for prymnesin-A2. Δ66 reflects a loss of a pentose unit. Prymnesin-A2 is singly glycosylated with 1x pentose unit. Right - MS/HRMS spectra of $[M+2H]^{2+}$ for prymnesin-A2 after CuAAC click coupling with **3**. Δ101.5 reflects addition of the coumarin moiety to the prymnesin toxin. Fragmentation of

monosaccharides is still visible after addition of **3**. Yellow star represents the activated fluorescent triazole product.

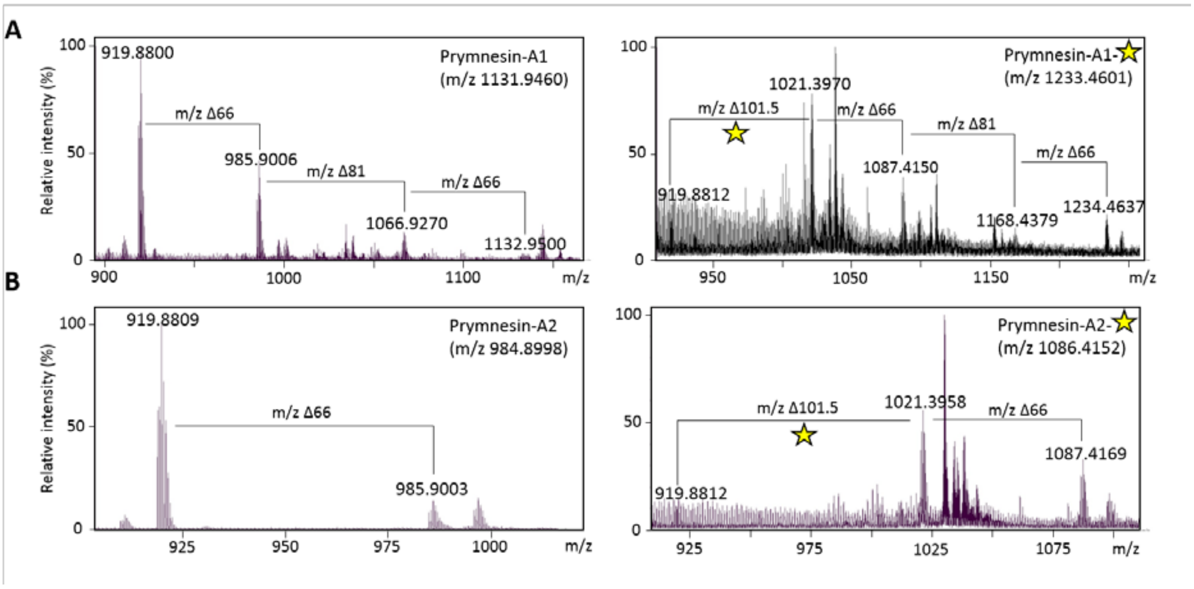


**Figure S4** – CuAAC click coupling of Prymnesin-A1 and -A2 from *P. parvum* 94A with 3-azido-7-hydroxycoumarin (**3**). (**A**) Left - MS/HRMS spectra of $[M+2H]^{2+}$ for prymnesin-A1. Δ66 reflects a loss of a pentose unit and Δ81 reflects a loss of a hexose unit. Prymnesin-A1 is triply glycosylated with 2x pentose units and 1x hexose. Right - MS/HRMS spectra of $[M+2H]^{2+}$ for prymnesin-A1 after CuAAC click coupling with **3**. (**B**) Left - MS/HRMS spectra of $[M+2H]^{2+}$ for prymnesin-A2. Δ66 reflects a loss of a pentose unit. Prymnesin-A2 is singly glycosylated with 1x pentose unit. Right - MS/HRMS spectra of $[M+2H]^{2+}$ for prymnesin-A2 after CuAAC click coupling with **3**. Δ101.5 reflects addition of the coumarin moiety to the prymnesin toxin. Fragmentation of monosaccharides is still visible after addition of **3**. Yellow star represents the activated fluorescent triazole product.

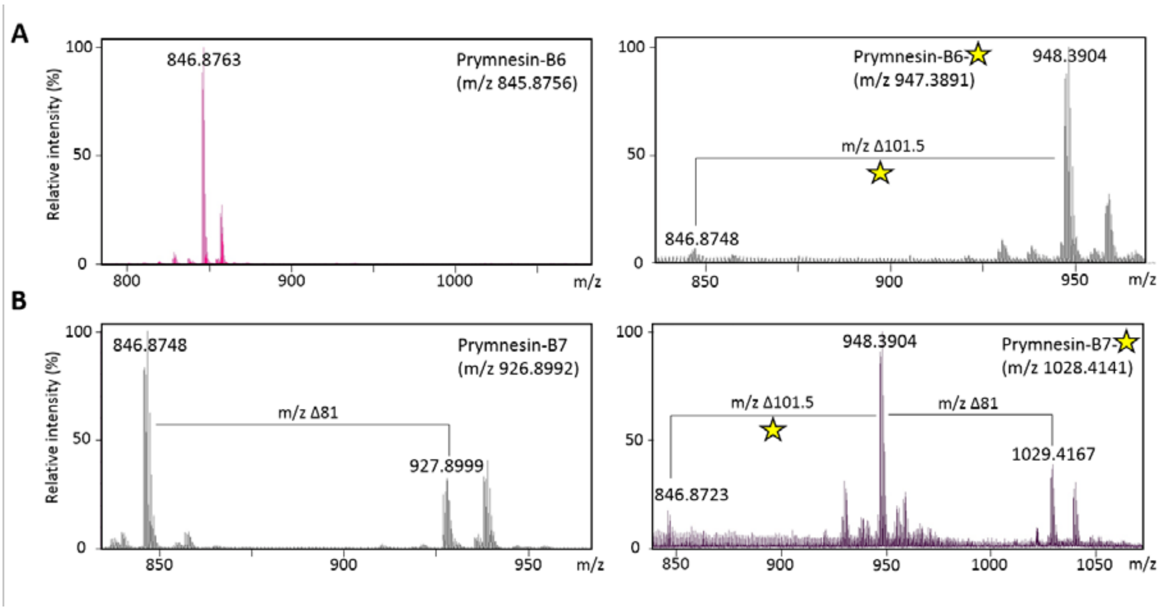


**Figure S5** – CuAAC click coupling of Prymnesin-B6 and -B7 from *P.* sp. 595 with 3-azido-7-hydroxycoumarin (**3**). (**A**) Left - MS/HRMS spectra of $[M+2H]^{2+}$ for prymnesin-B6. Prymnesin-B1 shows no fragmentation and is therefore thought to not be glycosylated. Right - MS/HRMS spectra of $[M+2H]^{2+}$ for prymnesin-B6 after CuAAC click coupling with **3**. (**B**) Left - MS/HRMS spectra of $[M+2H]^{2+}$ for prymnesin-B7. Δ81 reflects a loss of a hexose unit. Prymnesin-B7 is singly glycosylated with 1x hexose unit. Right - MS/HRMS spectra of $[M+2H]^{2+}$ for prymnesin-B7 after CuAAC click coupling with **3**. Δ101.5 reflects addition of the coumarin moiety to the prymnesin toxin. Fragmentation of monosaccharides is still visible after addition of **3**. Yellow star represents the activated fluorescent triazole product.

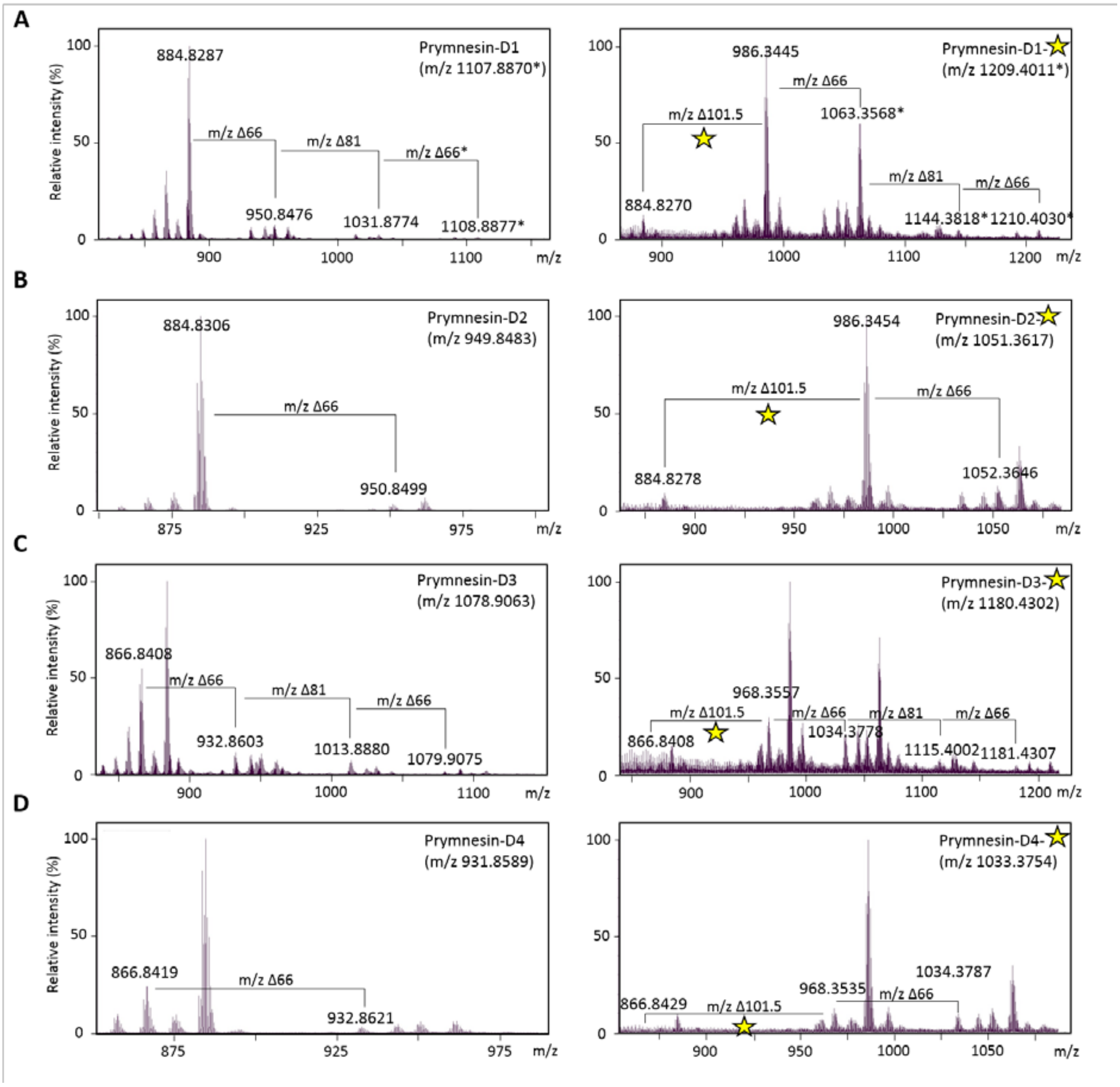


**Figure S6** – CuAAC click coupling of Prymnesin-D1, -D2, -D3 and -D4 from *P. patelliferum* 527D with 3-azido-7-hydroxycoumarin (**3**). (**A**) Left - MS/HRMS spectra of $[M +2H]^{2+}$ / $[M +Na +H]^{2+}$ for prymnesin-D1 ($[M +Na +H]^{2+}$ ions are indicated by '*'). Δ66 reflects a loss of a pentose unit and Δ81 reflects a loss of a hexose unit. Prymnesin-D1 is triply glycosylated with 2x pentose units and 1x hexose. Right - MS/HRMS spectra of $[M +2H]^{2+}$ / $[M + Na +H]^{2+}$ for prymnesin-D1 after CuAAC click coupling with **3** ($[M +Na +H]^{2+}$ ions are indicated by '*'). (**B**) Left - MS/HRMS spectra of $[M +2H]^{2+}$ for prymnesin-D2. Δ66 reflects a loss of a pentose unit. Prymnesin-D2 is singly glycosylated with 1x pentose unit. Right - MS/HRMS spectra of $[M +2H]^{2+}$ for prymnesin-D2 after CuAAC click coupling with **3**. (**C**) Left - MS/HRMS spectra of $[M +2H]^{2+}$ for prymnesin-D3. Δ66 reflects a loss of a pentose unit and Δ81 reflects a loss of a hexose unit. Prymnesin-D3 is triply glycosylated with 2x pentose units and 1x hexose. Right - MS/HRMS spectra of $[M +2H]^{2+}$ for prymnesin-D3 after CuAAC click coupling with **3**. (**D**) Left - MS/HRMS spectra of $[M +2H]^{2+}$ for prymnesin-D4. Δ66 reflects a loss of a pentose unit. Prymnesin-D4 is singly glycosylated with 1x pentose unit. Right - MS/HRMS spectra of $[M +2H]^{2+}$ for prymnesin-D2 after CuAAC click coupling with

**3**. Δ101.5 reflects addition of the coumarin moiety to the prymnesin toxin. Fragmentation of monosaccharides is still visible after addition of **3**. Yellow star represents the activated fluorescent triazole product.

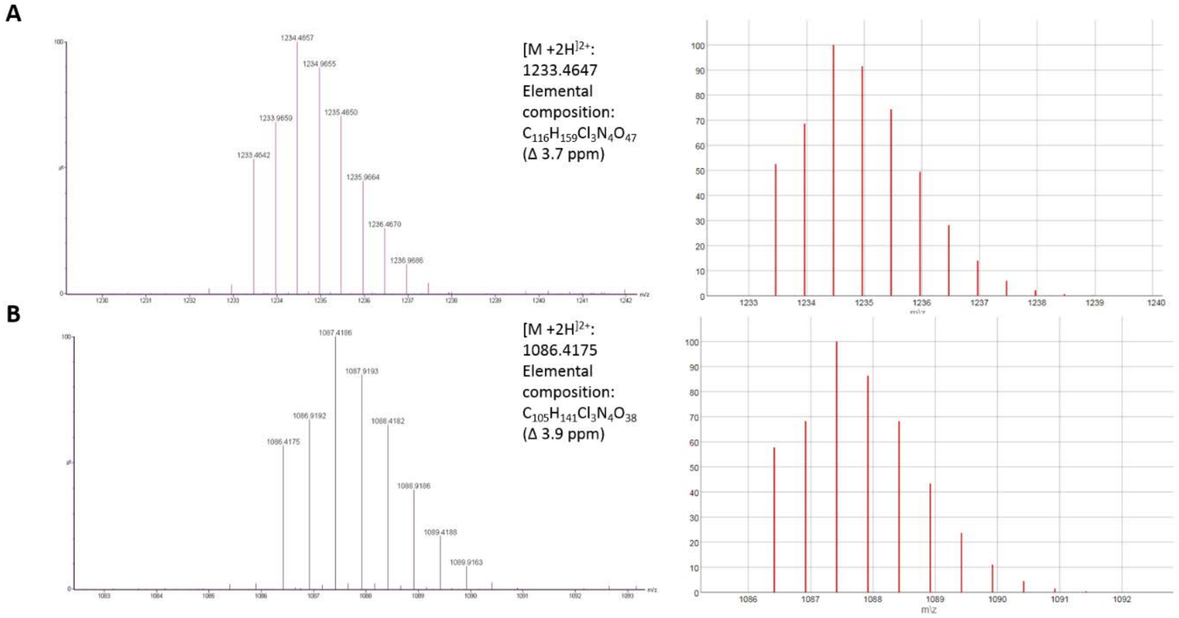


**Figure S7** – Comparison of measured vs theoretical isotope pattern signals for Prymnesin-A1 and -A2 from *P. parvum* 946/6. (**A**) Left- Measured isotope pattern distribution for Prymnesin-A1. Right – Theoretical isotope pattern distribution for $C_{116}H_{159}Cl_3N_4O_{47}$ calculated using enviPat[1]. (**B**) Left- Measured isotope pattern distribution for Prymnesin-A2. Right – Theoretical isotope pattern distribution for $C_{105}H_{141}Cl_3N_4O_{38}$ calculated using enviPat[1].

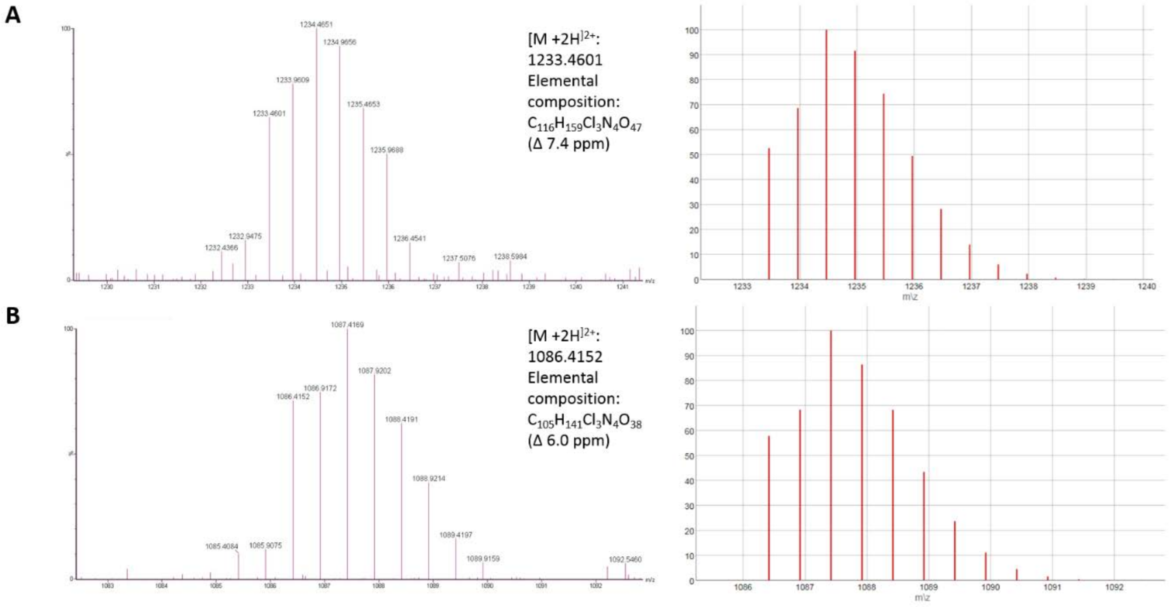


**Figure S8** – Comparison of measured vs theoretical isotope pattern signals for Prymnesin-A1 and -A2 from *P. parvum* 94A. (**A**) Left- Measured isotope pattern distribution for Prymnesin-A1. Right – Theoretical isotope pattern distribution for $C_{116}H_{159}Cl_3N_4O_{47}$ calculated using enviPat[1]. (**B**) Left- Measured isotope pattern

distribution for Prymnesin-A2. Right – Theoretical isotope pattern distribution for $C_{105}H_{141}Cl_3N_4O_{38}$ calculated using enviPat[1].

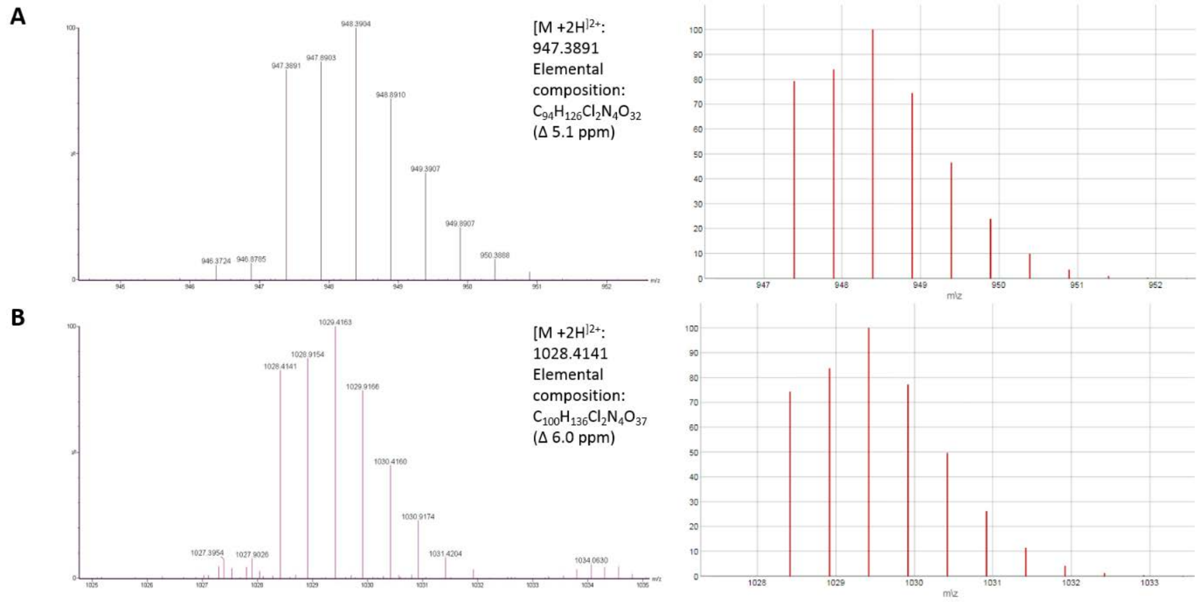


**Figure S9** – Comparison of measured vs theoretical isotope pattern signals for Prymnesin-B6 and -B7 from *P.* sp. 595. (**A**) Left- Measured isotope pattern distribution for Prymnesin-B6. Right – Theoretical isotope pattern distribution for $C_{94}H_{126}Cl_2N_4O_{32}$ calculated using enviPat[1]. (**B**) Left- Measured isotope pattern distribution for Prymnesin-B7. Right – Theoretical isotope pattern distribution for $C_{100}H_{136}Cl_2N_4O_{37}$ calculated using enviPat[1].

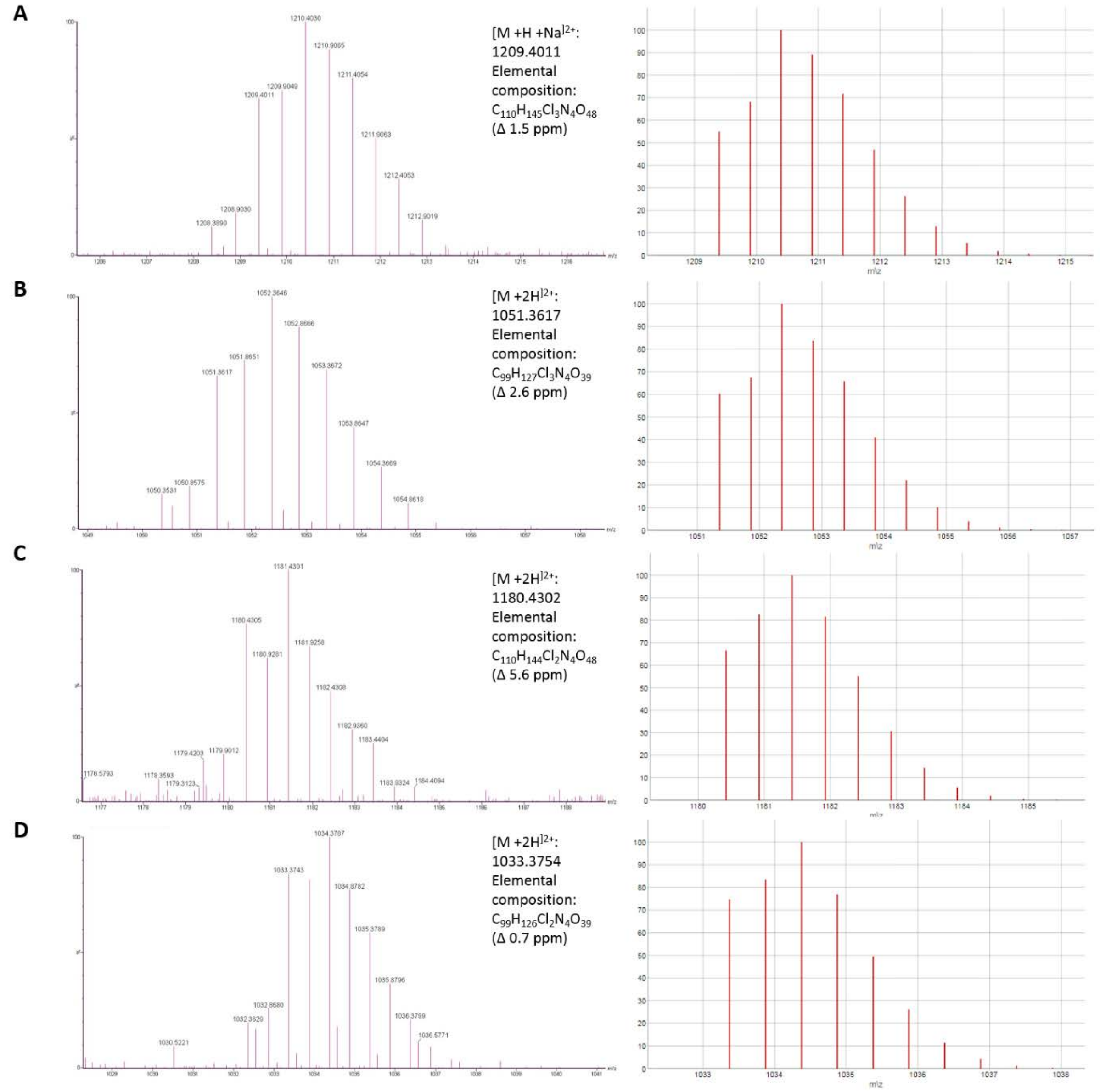


**Figure S10** – Comparison of measured vs theoretical isotope pattern signals for Prymnesin-D1, -D2, -D3 and -D4 from *P. patelliferum* 527D. (**A**) Left- Measured isotope pattern distribution for Prymnesin-D1. Right – Theoretical isotope pattern distribution for $C_{110}H_{145}Cl_3N_4O_{48}$ calculated using enviPat[1]. (**B**) Left- Measured isotope pattern distribution for Prymnesin-D2. Right – Theoretical isotope pattern distribution for $C_{99}H_{127}Cl_3N_4O_{39}$ calculated using enviPat[1]. (**C**) Left- Measured isotope pattern distribution for Prymnesin-D3. Right – Theoretical isotope pattern distribution for $C_{110}H_{144}Cl_2N_4O_{48}$ calculated using enviPat[1]. (**D**) Left- Measured isotope pattern distribution for Prymnesin-D4. Right – Theoretical isotope pattern distribution for $C_{99}H_{126}Cl_2N_4O_{39}$ calculated using enviPat[1].

## General experimental conditions

Reagents and anhydrous solvents were purchased from by Sigma Aldrich and were used without further purification. Analytical grade solvents were purchased from Fischer Scientific. Glassware was oven-dried and purged with nitrogen immediately before use, and reactions requiring inert atmosphere were run under $N_2$.

Reactions were monitored by thin-layer chromatography (TLC) on aluminium-backed, pre-coated silica gel plates (Silica Gel 60 F254, E. Merk) with the indicated eluents, and the TLC plates were visualised under UV light (λ 254 nm or 365 nm) and charring by dipping in ethanol-sulfuric acid (95:5, v/v) followed by heating. TLC of prymnesins was run on Analtech preparative uniplates (silica gel 1000 micron, 20 × 20 cm, cut to size). Flash column chromatography (FCC) was performed on a Biotage Horizon Isolera One using pre-packed SNAP ULTRA 25 µm silica gel cartridges.

NMR spectra were recorded using a Bruker Ultrashield Plus 400 MHz spectrometer at 298 K and analysed using TopSpin 4.0.1 software. Chemical shifts (δ) are reported in parts per million (ppm) and NMR assignments were made with the aid of COSY and HSQCed experiments.

Optical rotation values were measured using a Perkin Elmer® Model 341 Polarimeter at 20 °C at a wavelength of 589 nm (sodium D line) unless otherwise noted. Infrared spectra were recorded using a Perkin Elmer® SpectrumBX and UV-vis spectra using a Varian 50 Bio spectrometer.

For high resolution mass spectrometry (HR-MS) of synthetic samples, the compounds were diluted into methanol/water/formic acid (50/49.9/0.1) and infused into a Synapt G2-Si mass spectrometer (Waters, Manchester, UK) at 5-10 µL min$^{-1}$ using a Harvard Apparatus syringe pump. The mass spectrometer was controlled by MassLynx 4.1 software (Waters). It was operated in resolution and positive ion mode and calibrated using sodium formate. The following machine parameters were used for recording HRMS for synthetic compounds; the sample was analysed for 2 min with 1 s MS scan time over the range of 50-1200 m/z with 3.5 kV capillary voltage, 40 V cone voltage, 120°C cone temperature. Leu-enkephalin peptide (0.25 µM, Waters) was infused at 10 µL min$^{-1}$ as a lock mass (m/z 556.2766) and measured every 10 s. Spectra were generated in MassLynx 4.1 by combining a number of scans, and peaks were centred using automatic peak detection with lock mass correction.

## Extraction of prymnesin toxins from *P. parvum* cell cultures

Prymnesin toxin extractions were performed using a modified protocol of Manning & La Claire[2]. In brief, cultures of Prymnesium (150 mL) were grown in 5PSU f/2 media at room temperature in a 14/10 h (light/dark) cycle as previously described[3]. After 3 weeks, the cells were harvested by centrifugation (4000 × g for 5 minutes) and the supernatant discarded. The cells were suspended in cold acetone (2 mL, -20 °C) and subject to vortex mixing for two minutes. The resulting suspensions were then centrifuged at 30,000 × g for 5 minutes. The supernatant was discarded, being careful not to disturb the cell debris, and the pellets were subject twice more to the same acetone wash. The cell pellets were then suspended in MeOH (2 mL) and vortex mixed for two minutes, after which time the cell debris was pelleted by centrifugation (30,000 × g for 5 minutes) and the supernatant was collected. This methanol extraction was repeated twice more, followed by three rounds of analogous extraction using *n*-PrOH. The MeOH and *n*-PrOH extracts were combined, dried *in vacuo,* and stored at -20 °C until further use.

## CuAAC coupling of prymnesin extracts

Dried Prymnesium cell extracts were resuspended in 1 mL of 50% aqueous EtOH. An excess of 3-azido-7-hydroxycoumarin (500 µg in 200 µL of 50% EtOH) was added to the prymnesin extracts. A freshly prepared solution of 0.1M aqueous copper sulfate and 0.2M aqueous sodium ascorbate (10 µL) was added to start the coupling reaction. The reaction was left at room temperature in the dark overnight. To follow reaction progress, thin layer chromatography analysis was carried out following solvent conditions described by Manning and La Claire[2]. The TLC plate was allowed to dry in a fume hood for 30 minutes before being visualized by long wave UV (λ 365 nm) (Fig. S6). The clicked extracts were dried in *vacuo* and stored at -20 °C until further use.

## LC-MS detection of prymnesin toxins

The Prymnesium extracts were analysed by LC-MS on a Synapt G2-Si mass spectrometer coupled to an Acquity UPLC system (Waters, Manchester, UK). The extracts were first dissolved into 1000 µL of 50% EtOH. Aliquots of 7 µL sample were injected onto an Acquity UPLC® BEH C18 column, 1.7 µm, 1x100 mm (Waters) and eluted with a gradient of 1-60% acetonitrile in 0.1% formic acid in 6 min at a flow rate of 0.08 ml $min^{-1}$ with a column temperature of 45°C. The mass spectrometer was controlled using Masslynx 4.1 software (Waters) and operated in positive MS-Tof and resolution mode with a capillary voltage of 3 kV and a cone voltage of 40 V in the m/z range of 100-2000. Leu-enkephalin peptide (0.25 µM, Waters) was infused at 10 µl $min^{-1}$ as a lock mass and measured every 30 s.

## Chemistry

### (Bromoethynyl) triethylsilane[4]

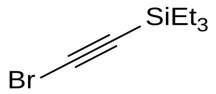


Triethylsilyl ethyne (1.0 mL, 5.58 mmol) and $AgNO_3$ (190 mg, 1.1 mmol) were dissolved into dry acetone (30 mL) under $N_2$. The reaction mixture was stirred vigorously in the dark for 20 minutes after which time the reaction had turned a milky white colour. *N*-Bromosuccinimide (1.1g, 6.1 mmol) was added and the reaction was stirred for a further 3 hours in the dark. TLC (neat hexane) showed complete consumption of the starting material and the reaction mixture was filtered and the solvent was removed under reduced pressure. The crude mixture was purified on a short silica column using pure n-hexane to give (bromoethynyl) triethylsilane (1.0 g, 84%) as a colourless oil; $R_f$ 0.84 (neat hexane); $\delta_H$(400 MHz; $CDCl_3$) 0.99 (t, *J* = 8.1 Hz, 9H, $Si(CH_2\underline{CH_3})_3$), 0.61 (q, *J* = 8.1 Hz, 6H, $Si(C\underline{H_2}CH_3)_3$); $\delta_C$ (101 MHz; $CDCl_3$) 84.6 (Si-C≡$\underline{C}$-Br), 61.6 (Si-$\underline{C}$≡C-Br), 7.3 ($Si(CH_2\underline{C}H_3)_3$), 4.3 ($Si(\underline{C}H_2CH_3)_3$). The $^1$H and $^{13}$C NMR data were in accordance with the literature[4].

### Pent-4-yn-1-yl 2',3',4',6'-tetra-O-acetyl-β-D-galactopyranoside (5)[5]

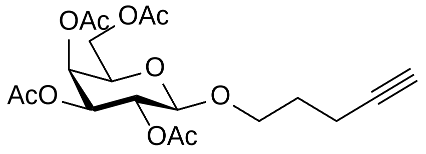


β-D-Galactose pentaacetate (**4**) (1.0 g, 2.6 mmol) and 4-pentyn-1-ol (1 mL, 10.7 mmol) were dissolved in anhydrous DCM (20 mL) and the solution was cooled to 0 °C. $BF_3.Et_2O$ (2 mL, 16 mmol) was slowly added and the reaction mixture was stirred overnight at room temperature. TLC (hexane/EtOAc 8:2) showed complete consumption of the donor and the reaction was quenched with sat. aqueous $NaHCO_3$ (20 mL). The reaction mixture was extracted with DCM (3 × 10 mL) and the organic layers were combined and dried over $MgSO_4$, before being filtered and concentrated *in vacuo* to give crude product which was purified by FCC to give the title compound (**5**) (450 mg, 71%) as a yellow oil. $R_f$ 0.1 (hexane/EtOAc 8:2); $[\alpha]_D$ +2.8 (*c* 1.0 $CHCl_3$); $\delta_H$(400 MHz; $CDCl_3$) 5.40 (dd, $J_{3',4'}$ = 2.9 Hz, $J_{4',5'}$ = 1.1 Hz, 1H, H-4'), 5.21 (dd, $J_{1',2'}$ = 8.0 Hz, $J_{2',3'}$ = 10.5 Hz, 1H, H-2'), 5.03 ($J_{2',3'}$ = 10.5 Hz, $J_{3',4'}$ = 2.9 Hz, 1H, H-3'), 4.47 (d, $J_{1',2'}$ = 8.0 Hz, 1H, H-1'), 4.22 – 4.11 (m, 2H, H-6'a,b), 3.99 (dt, $^2J_{1a,1b}$ = 9.7 Hz, H-1a, $J_{1a,2a}$ = 5.2 Hz, 1H, H-1a), 3.93-3.90 (m, 1H, H-5') 3.67-3.61 (m, 1H, H-1b), 2.29-2.25 (m, 2H, H-3), 2.16 (s, 3H, OAc), 2.08 (s, 3H, OAc), 2.06 (s, 3H, OAc), 1.99 (s, 3H, OAc), 1.95 (t, $^4J_{3,5}$ = 2.7 Hz, 1H, H-5), 1.89-1.71 (2H, m,

2a,b); $\delta_C$(101 MHz; $CDCl_3$) 170.4, 170.3, 170.2, 169.5 (4 × C=O), 101.6 (C1'), 83.4 (C4), 70.9 (C3'), 70.6 (C5'), 68.9 (C2'), 68.8 (C5), 68.3 (C1), 67.0 (C4'), 61.3 (C6'), 28.2 (C2), 20.7, 20.7, 20.6, 20.6 (4 × Ac), 14.8 (C3); HRMS (ESI$^+$) *m/z* calc. for $C_{19}H_{26}O_{10}Na^+$ 437.1418 ([M.Na]$^+$) found 437.1421 [M.Na]$^+$. $^1$H and $^{13}$C NMR values were in agreement with literature values[5].

## Pent-4-yn-1-yl β-D-galactoside (1)[5]

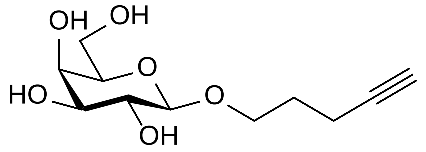


A piece of sodium metal (5 mg, 0.2 mmol) was placed in dry MeOH (10 mL). After the cessation of effervescence, the solution of sodium methoxide was added by syringe to a solution of pent-4-yn-1-yl 2',3',4',6'-tetra-O-acetyl-β- D-galactopyranoside (**5**) (750 mg, 1.8 mmol) in MeOH (10 mL) under $N_2$ and the reaction mixture was left to stir overnight. Low resolution mass spectrometry showed only the desired product, and the reaction mixture was neutralised to pH 7.0 with Amberlite® 120 H+ resin, before being filtered and concentrated under reduced pressure to give the title compound (**1**) (370 mg, 83%) as a yellow oil; $[\alpha]_D$ -13.0 (*c* = 1.0, MeOH) (lit[5]. $[\alpha]_D^{26}$ -24.6 (*c* = 0.85, MeOH)); $\delta_H$(400 MHz; $CD_3OD$) 4.23 (d, $J_{1',2'}$ = 7.2 Hz, 1H, H-1'), 3.98 (dt, $^2J_{1a,1b}$ = 12.4 Hz, $J_{1a,2}$ = 6.3 Hz, 1H, H-1a), 3.86 (dd, $J_{3',4'}$ = 3.1 Hz, $J_{4',5'}$ = 1.0 Hz, 1H, H-4'), 3.77-3.75 (m, 2H, H-6'a,b), 3.67 (dt, $^2J_{1a,1b}$ = 12.4 Hz, $J_{1a,2}$ = 6.3 Hz, 1H, H-1b), 3.55-3.46 (m, 3H, H-2',3',5'), 2.35-2.30 (m, 2H, H-3), 2.22 ($^4J_{3,5}$ = 2.8, 1H, H-5), 1.86-1.81 (m, 1H, H-2); $\delta_C$(101 MHz; $CD_3OD$) 103.7 (C1'), 83.4 (C4), 75.2 (C3'), 73.6 (C5'), 71.2 (C2'), 68.9 (C4'), 68.2 (C5), 67.9 (C1), 61.1 (C6'), 28.7 (C2), 14.4 (C3); HRMS (ESI$^+$) *m/z* calc. for $C_{11}H_{18}O_6Na^+$ 269.0996 ([M.Na]$^+$) found 269.0990 [M.Na]$^+$. The $^1$H and $^{13}$C NMR spectra were in agreement with literature values[5].

## 7-(Triethylsilyl)hepta-4,6-diyn-yl-β-D-galactoside (6)

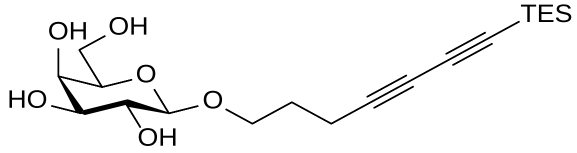


4-Pentyn-yl-β-D-galactoside (**1**) (370 mg, 1.5 mmol), $BuNH_2$ (7 mL), $H_2O$ (3 mL) and $NH_2OH.HCl$ (35 mg, 0.5 mmol) were dissolved into MeOH (20 mL). The reaction mixture was cooled to 0 °C and CuCl (15 mg, 0.15 mmol) was added in a single portion. (Bromoethynyl)(triethyl)silane (1.0 g, 4.9 mmol) was added dropwise by syringe. The reaction immediately went bright orange, and after stirring at 0 °C for 1 hour the reaction was a dark blood red. The reaction mixture was diluted with EtOAc (50 mL)

and washed with sat. aqueous $NH_4Cl$ solution (3 × 10 mL). The aqueous layer was then extracted with EtOAc (3 × 5 mL) and the organic layers were combined, dried over $MgSO_4$, filtered and concentrated *in vacuo*. The crude residue was purified by FCC (gradient of 0% to 20% MeOH in DCM) to return the title compound **6** (135 mg, 23%) as a yellow powder; $R_f$ 0.44 (DCM/MeOH 9:1); $[\alpha]_D$ = +10 (*c* 1.0, MeOH); $\delta_H$(400 MHz; $CD_3OD$) 4.12 (d, $J_{1',2'}$ = 7.2 Hz, 1H, H-1'), 3.86 (dt, $^2J_{1a,1b}$ = 10.1 Hz, $J_{1a,2}$ = 4.1 Hz, 1H, H-1a), 3.74 (dd, $J_{3',4'}$ = 3.1 Hz, $J_{4',5'}$ = 1.0 Hz, 1H, H-4'), 3.68-3.60 (m, 2H, H-6'a,b), 3.55 (dt, $^2J_{1a,1b}$ = 10.1 Hz, $J_{1b,2}$ = 4.1 Hz, 1H, H-1b), 3.43-3.35 (m, 3H, H-2',3',5'), 2.36 (t, $J_{2,3}$ = 7.1 Hz, 2H, H-3), 1.77-1.71 (m, 2H, H-2), 0.91 (t, $J_{8,9}$ = 8.0 Hz, 9H, H-9), 0.52 (q, $J_{8,9}$ = 8.0 Hz, 6H, H-8) $\delta_C$(101 MHz; $CD_3OD$) 101.3 (C1'), 87.1 (C6), 77.2 (C4), 76.18 (C7), 72.8 (C3'), 71.2 (C5'), 68.8 (C2'), 66.5 (C4'), 65.4 (C1), 62.8 (C5), 58.7 (C6'), 26.0 (C2), 12.8 (C3) 3.9 ($CH_3$), 1.4 ($CH_2$); HRMS (ESI$^+$) *m/z* calc for $C_{19}H_{32}O_6SiNa^+$ 407.1861 ($[M.Na]^+$), found 407.1859 $[M.Na]^+$.

## Hepta-4,6-diyn-yl-β-D-galactoside (2)

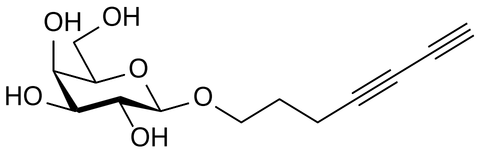


To a solution of 7-(triethylsilyl)hepta-4,6-diyn-yl-β-D-galactoside (**6**) (34 mg, 90 µmol) in anhydrous THF (3 mL) was added $Et_3N.3HF$ (145 µL, 0.9 mmol). The reaction was stirred over night at room temperature, after which time TLC (DCM/MeOH 9:1) showed the reaction had gone to completion. The crude mixture was dried under reduced pressure and purified by FCC to give the title compound **2** (20 mg, 88%) as a pink powder. $R_f$ 0.16 (DCM/MeOH 9:1); $[\alpha]_D$ = -13.0 (*c* = 1.0, MeOH); $\delta_H$(400 MHz; $CD_3OD$) 4.11 (d, $J_{1',2'}$ = 7.1 Hz, 1H, H-1'), 3.85 (dt, $^2J_{1a,1b}$ = 10.2 Hz, $J_{1a,2}$ = 6.0 Hz, 1H, H-1a), 3.73 (dd, $J_{3',4'}$ = 3.1 Hz, $J_{4',5'}$ = 1.0 Hz, 1H, H-4'), 3.65-3.63 (m, 2H, H-6'a,b) 3.54 (dt, $^2J_{1a,1b}$ = 10.2 Hz, $J_{1a,2}$ = 6.0 Hz, 1H, H-1b), 3.43-3.34 (m, 3H, H-2',3',5'), 2.41 (t, $^6J_{3,7}$ = 1.2 Hz, 1H, H-7), 2.33 (t, $J_{2,3}$ = 7.1 Hz, 2H, H-3), 1.77-1.70 (m, 2H, H-2); $\delta_C$(101 MHz; $CD_3OD$) 103.7 (C1'), 76.8 (C4), 75.2 (C3'), 73.6 (C5'), 71.2 (C2'), 68.9 (C4'), 67.7 (C1), 67.6 (C6), 65.0 (C7), 64.4 (C5), 61.0 (C6'), 28.3 (C3), 14.9 (C2); HRMS (ESI$^+$) *m/z* for $C_{13}H_{18}O_6Na^+$ 293.0996 ($[M.Na]^+$), found 293.0995 $[M.Na]^+$.

## 3-azido-7-hydroxycoumarin (3)[7]

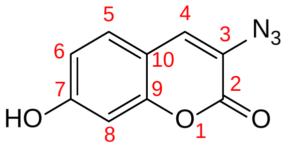

A suspension of 2,4-hydroxy benzaldehyde (4.2 g, 30 mmol), *N*-acetylglycine (3.5 g, 30 mmol) and sodium acetate (12.5 g, 150 mmol) in acetic anhydride (100 mL) was heated to reflux overnight. The reaction mixture was poured onto ice and the resulting yellow solid was filtered off and dissolved in a 1:1 mixture of EtOH/HCl (conc.) (50 mL) and heated to reflux for 1 hour. The resulting mixture was then cooled in an ice bath and $NaNO_2$ (4.2 g, 61 mmol) in ice cold water (40 mL) was added in a single portion. After 10 minutes $NaN_3$ (6.5 g, 100 mmol) was added in small portions and the reaction was stirred for 1 hour. The resulting precipitate was filtered off, washed with ice cold water and dried *in vacuo* before being purified by FCC to give crude 3-azido-7-hydroxycoumarin (**3**) (410 mg, 7%) as a brown powder. $R_f$ 0.53 (hexane/EtOAc 6:4); $\nu_{max}/cm^{-1}$ (ATR-IR) 3292 (br, OH), 2107 (s, $N_3$), 1676 (m, C=O), 1621, 1594, 1342 (m, Ar-O), 1317 (s, C=O), 1258, 1220, 1120, 926, 836, 624; $\delta_H$(400 MHz; DMSO-$d_6$) 10.53 (s, 1H, OH), 7.61 (s, 1H, H-4), 7.49 (d, $J_{5,6}$ = 8.5 Hz, 1H, H-5), 6.82 (dd, $^4J_{6,8}$ = 2.2 Hz, $J_{5,6}$ = 8.5 Hz, 1H, H-6), 6.76 (d, $^4J_{6,8}$ = 2.2 Hz, 1H, H-8); $\delta_C$(101 MHz; DMSO-$d_6$) 160.7 (C2), 157.8 (C7), 153.2 (C9), 129.6 (C4), 128.3 (C5), 121.6 (C3), 114.3 (C10), 111.8 (C6), 102.5 (C8); The $^1$H NMR data were in accordance with the literature[7].

## 3-[1-(7-Hydroxy-coumarin)-1H-1,2,3-triazol-4-yl] propyl β-D-galactopyranoside (7)

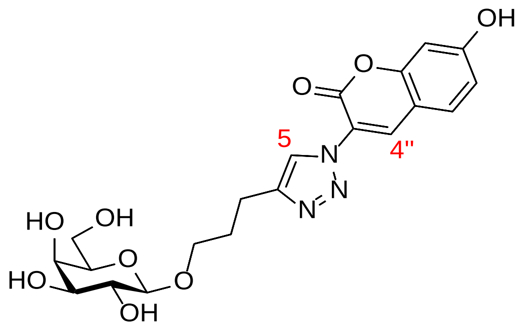


4-Pentyn-yl-β-D-galactoside (**1**) (25 mg, 0.1 mmol) and 3-azido-7-hydroxycoumarin (**3**) (32 mg, 0.1 mmol) were dissolved in MeOH/$H_2O$ (1:1) (2 mL). The reaction was started by adding 1M aqueous copper sulfate solution (20 µL) and 1M aqueous sodium ascorbate solution (50 µL) and then stirred at room temperature for 2 hours before being concentrated *in vacuo*. The crude residue was purified by semi-prep TLC ($CHCl_3$/MeOH 85:15) to give the title compound **7** as a yellow powder (25 mg, 56%); $R_f$ 0.53 ($CHCl_3$/MeOH 85:15); $[\alpha]_D$ -12.6 (*c* = 0.5, MeOH); UV-vis (DMSO) $\lambda_{abs}$ = 393 nm; $\delta_H$(400 MHz; $CD_3OD$) 8.36 (s, 1H, H-5), 8.27 (1H, s, H-4''), 7.54 (d, $J_{5'',6''}$ = 8.8 Hz, 1H, H-5''), 6.80 (dd, $^4J_{6'',8''}$ = 2.4 Hz, $J_{5'',6''}$ = 8.8 Hz, 1H, H-6''), 6.72 (d, $^4J_{6'',8''}$ = 2.4 Hz, 1H, H-8''), 4.14 (d, $J_{1',2'}$ = 7.6 Hz, 1H, H-1'), 3.87 (dt, $J_{1a,1b}$ = 9.9 Hz, $J_{1a,2}$ = 6.3 Hz, 1H, H-1a), 3.74 (dd, $J_{3',4'}$ = 3.1 Hz, $J_{4',5'}$ = 1.0 Hz, H-4'), 3.69-3.62 (m, 2H, H-6'a,b), 3.52 (dt, $J_{1a,1b}$ = 9.9 Hz, $J_{1b,2}$ = 6.3 Hz, 1H, H-1b), 3.48-3.36 (m, 3H, H-5',2',3'), 2.83 (t, $J_{2,3}$ = 7.4 Hz, 2H, H-3), 1.96-1.90 (m, 2H, H-2); $\delta_C$(101 MHz; DMSO) 156.9 (C7''), 155.2 (C2''), 147.2 (Ar), 136.7 (C5), 131.3 (C5''), 123.5 (C4''), 115.0 (C6''), 104.0 (C1'), 102.7 (C8''), 75.6 (C5'), 73.8 (C2'), 71.0 (C3'), 68.7

(C4’), 68.1 (C1), 60.9 (C6’), 29.6 (C2), 22.0 (C3); HRMS (ESI$^+$) *m/z* calcd. for $C_{20}H_{24}N_3O_9^+$ 450.1507 ([M.H]$^+$) found 450.1505 [M.H]$^+$.

## 5-[1-(7-Hydroxy-coumarin)-1H-1,2,3-triazol-4-yl] pent-4-yn-1-yl β-ᴅ-galactopyranoside (8)

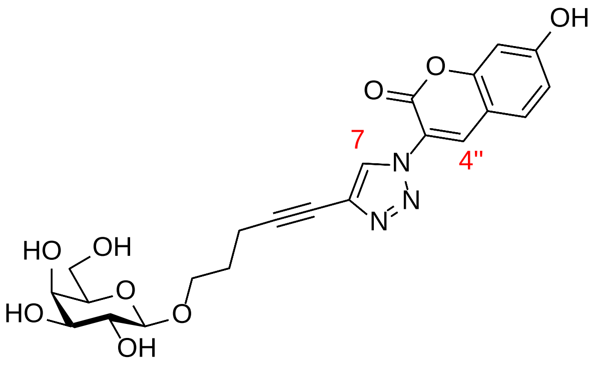


Hepta-4,6-diyn-yl-β-ᴅ-galactoside (**2**) (27 mg, 0.1 mmol) and 3-azido-7-hydroxycoumarin (**3**) (32 mg, 0.1 mmol) were dissolved in MeOH/$H_2O$ (1:1) (2 mL). The reaction was started by adding 1M aqueous copper sulfate solution (20 µL) and 1M aqueous sodium ascorbate solution (50 µL) and then stirred at room temperature for 2 hours before being concentrated *in vacuo*. The crude residue was purified by semi-prep TLC ($CHCl_3$/MeOH 85:15) to give the title compound (**8**) as a yellow powder (25 mg, 53%); $R_f$ 0.72 ($CHCl_3$/MeOH 85:15); $[\alpha]_D$ = -1.0 (*c* = 1.0, MeOH), $[\alpha]_{436}$ = -27.4 (*c* = 1.0, MeOH); UV-vis (DMSO) $\lambda_{abs}$ = 349 nm; $\delta_H$(400 MHz; $CD_3OD$) 8.49 (s, 1H, H-7), 8.41 (1H, s, H-4’’), 7.55 (d, $J_{5'',6''}$ = 8.3 Hz, 1H, H-5’’), 6.80 (dd, $^4J_{6'',8''}$ = 2.4 Hz, $J_{5'',6''}$ = 8.3 Hz, 1H, H-6’’), 6.73 (d, $J_{6'',8''}$ = 2.4 Hz, 1H, H-8’’), 4.15 (d, $J_{1',2'}$ = 7.5 Hz, 1H, H-1’), 3.99-3.92 (m, 1H, H-1), 3.74 (dd, $J_{3',4'}$ = 3.2 Hz, $J_{4',5'}$ = 1.0 Hz, H-4’), 3.70-3.60 (m, 4H, H-1b,5’,2’,3’), 3.46-3.36 (m, 2H, 6’a, 6’b) 2.53 (t, $J_{2,3}$ = 7.4 Hz, 2H, H-3), 1.88-1.81 (m, 2H, H-2); $\delta_C$(101 MHz; DMSO) 156.8 (C2’’), 137.9 (C4’’), 131.4 (C5’’), 130.4 (Ar), 128.4 (C7), 115.4 (C6’’), 104.0 (C1’), 102.8 (C8’’), 94.4 (C4), 75.6 (C5’), 73.9 (C2’), 71.1 (C3’), 70.5 (C5), 68.6 (C4’), 67.6 (C1), 60.9 (C6), 28.8 (C2), 15.9 (C3); HRMS (ESI$^+$) *m/z* calcd. for $C_{22}H_{24}N_3O_9^+$ 474.1507 ([M.H]$^+$) found 474.1510 [M.H]$^+$.

## NMR Spectra:

(Bromoethynyl) triethylsilane

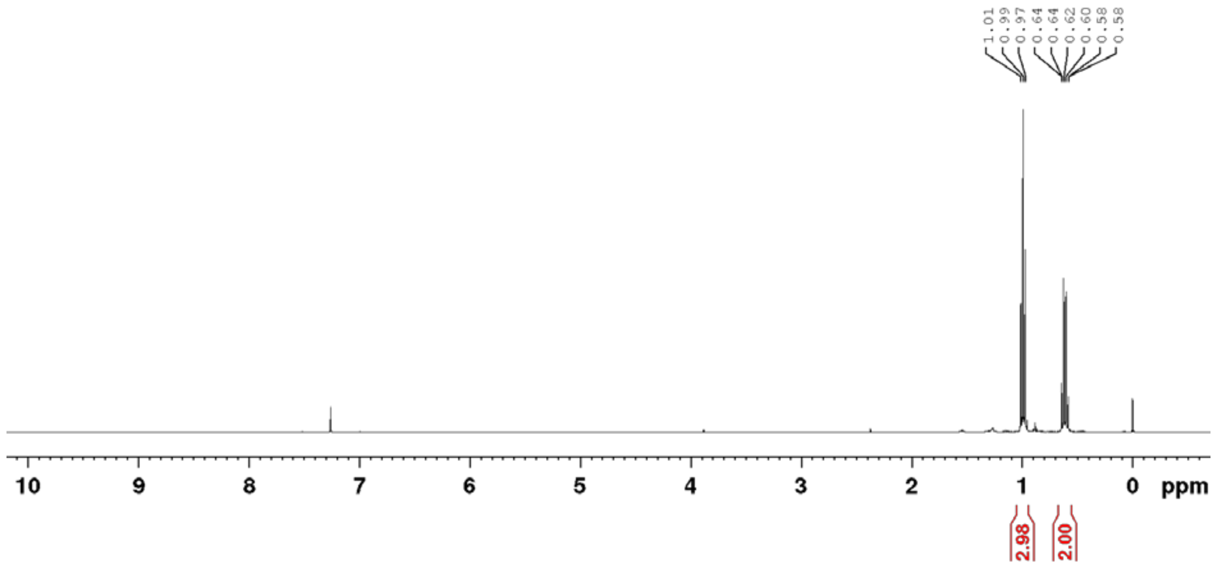


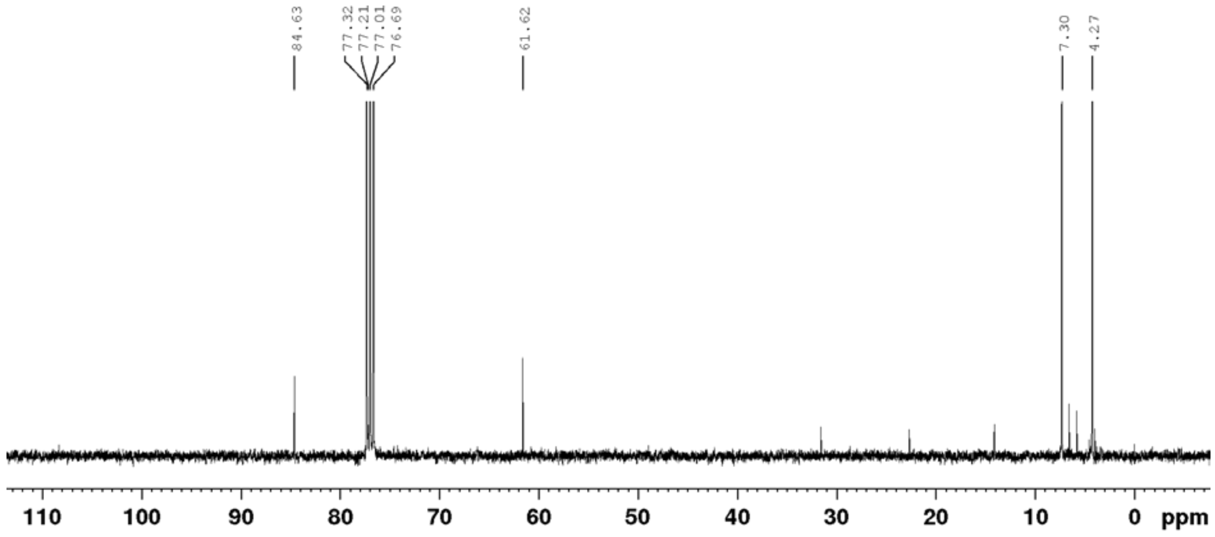

Pent-4-yn-1-yl 2’,3’,4’,6’-tetra-O-acetyl-β-ᴅ-galactopyranoside (5)

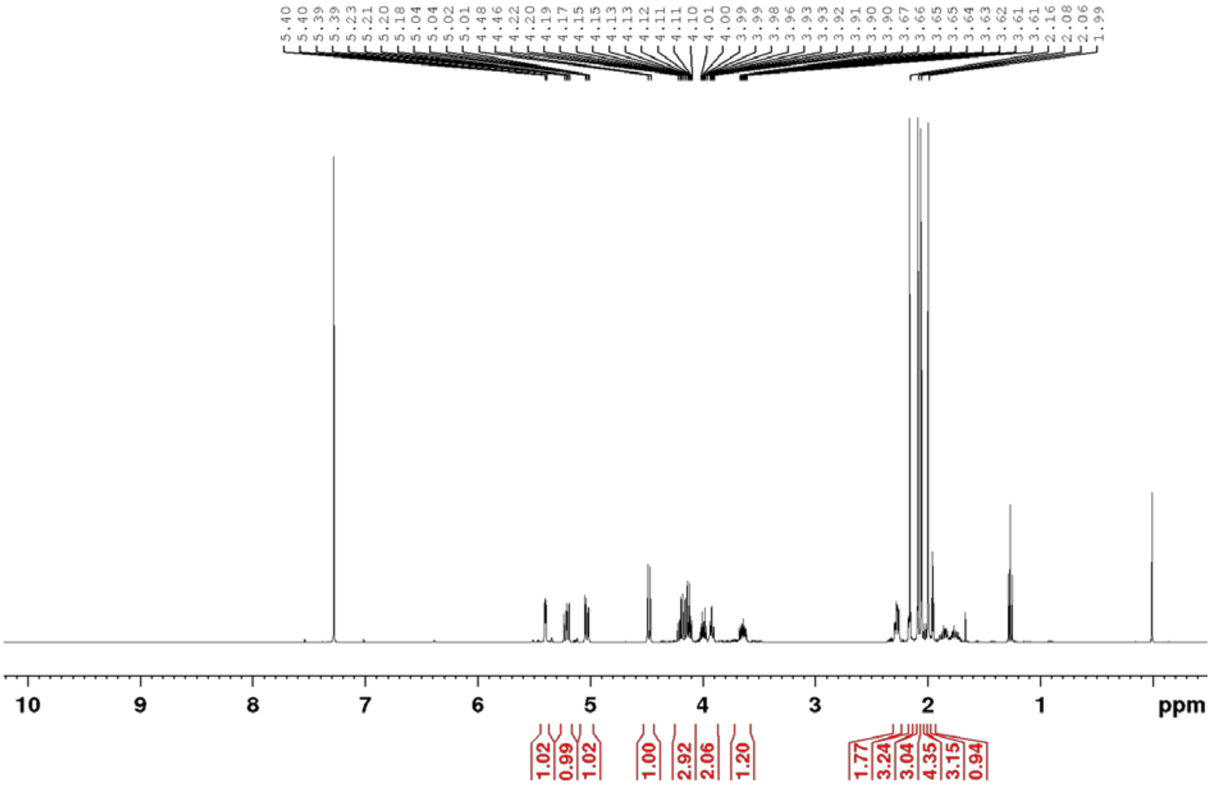


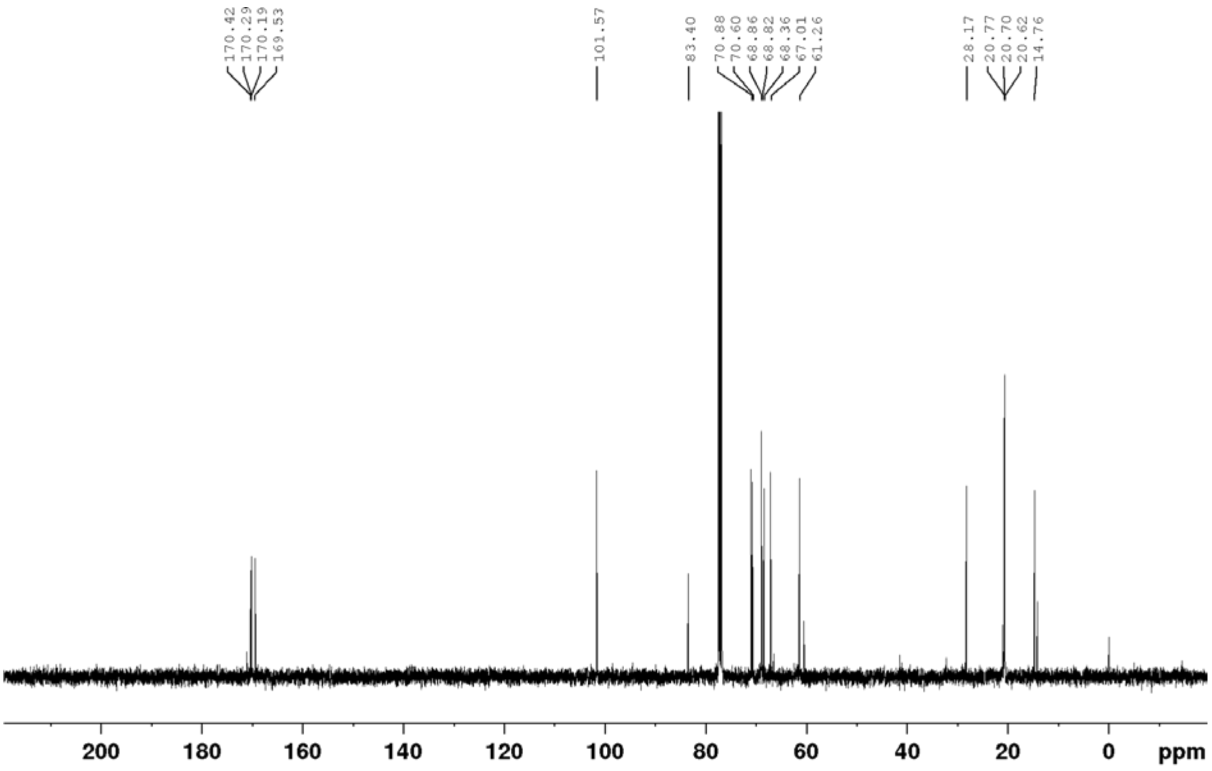

Pent-4-yn-1-yl β-ᴅ-galactoside (1)

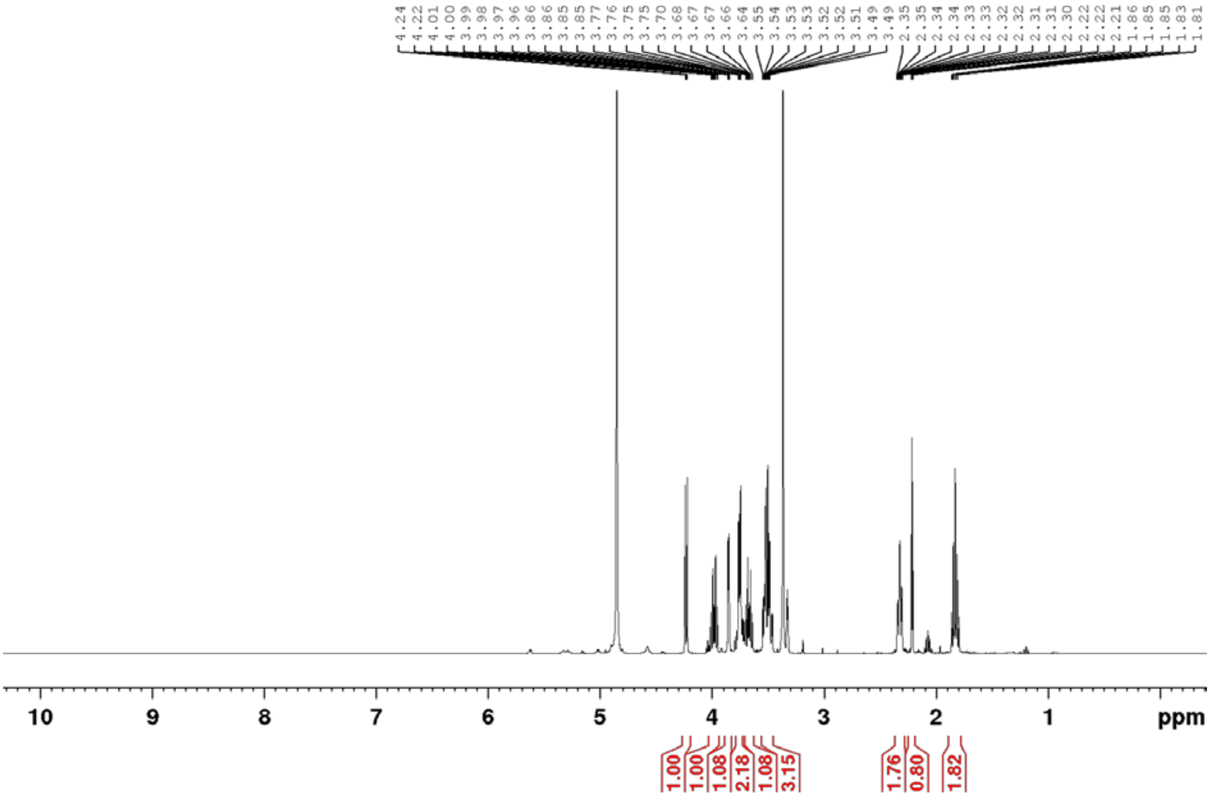

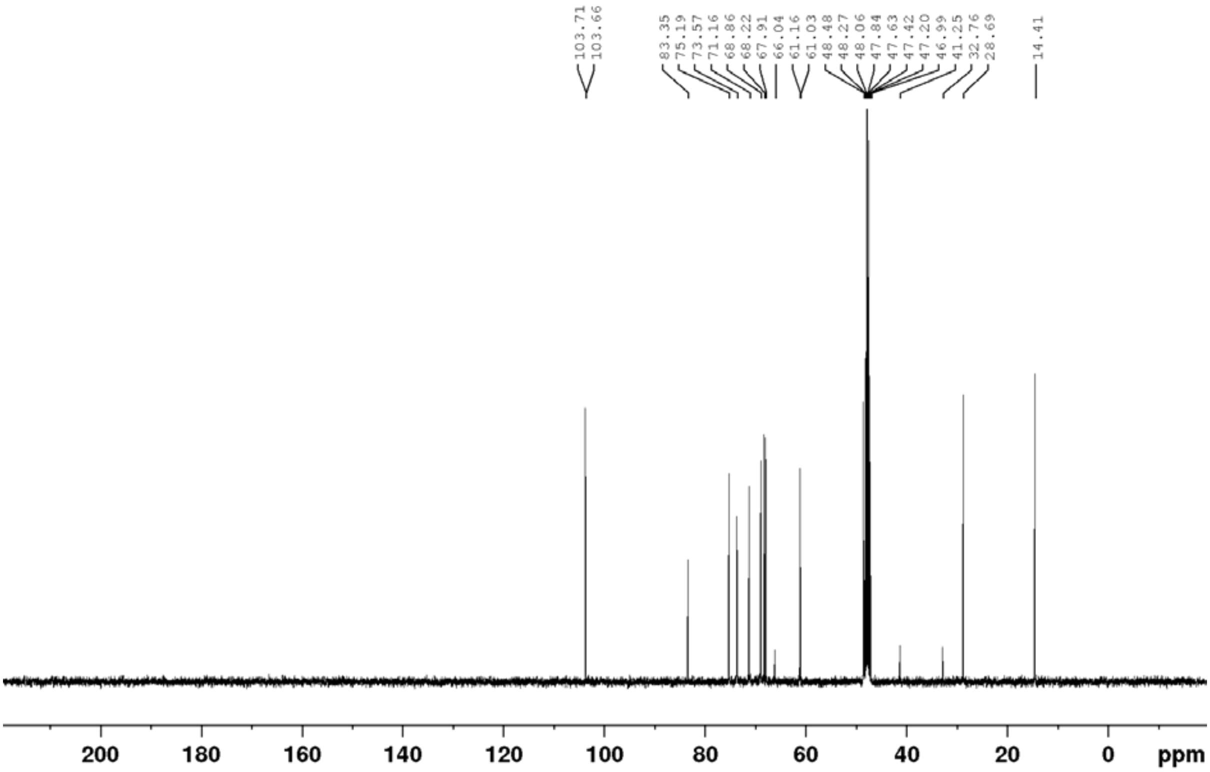

7-(Triethylsilyl)hepta-4,6-diyn-yl-β-ᴅ-galactoside (6)

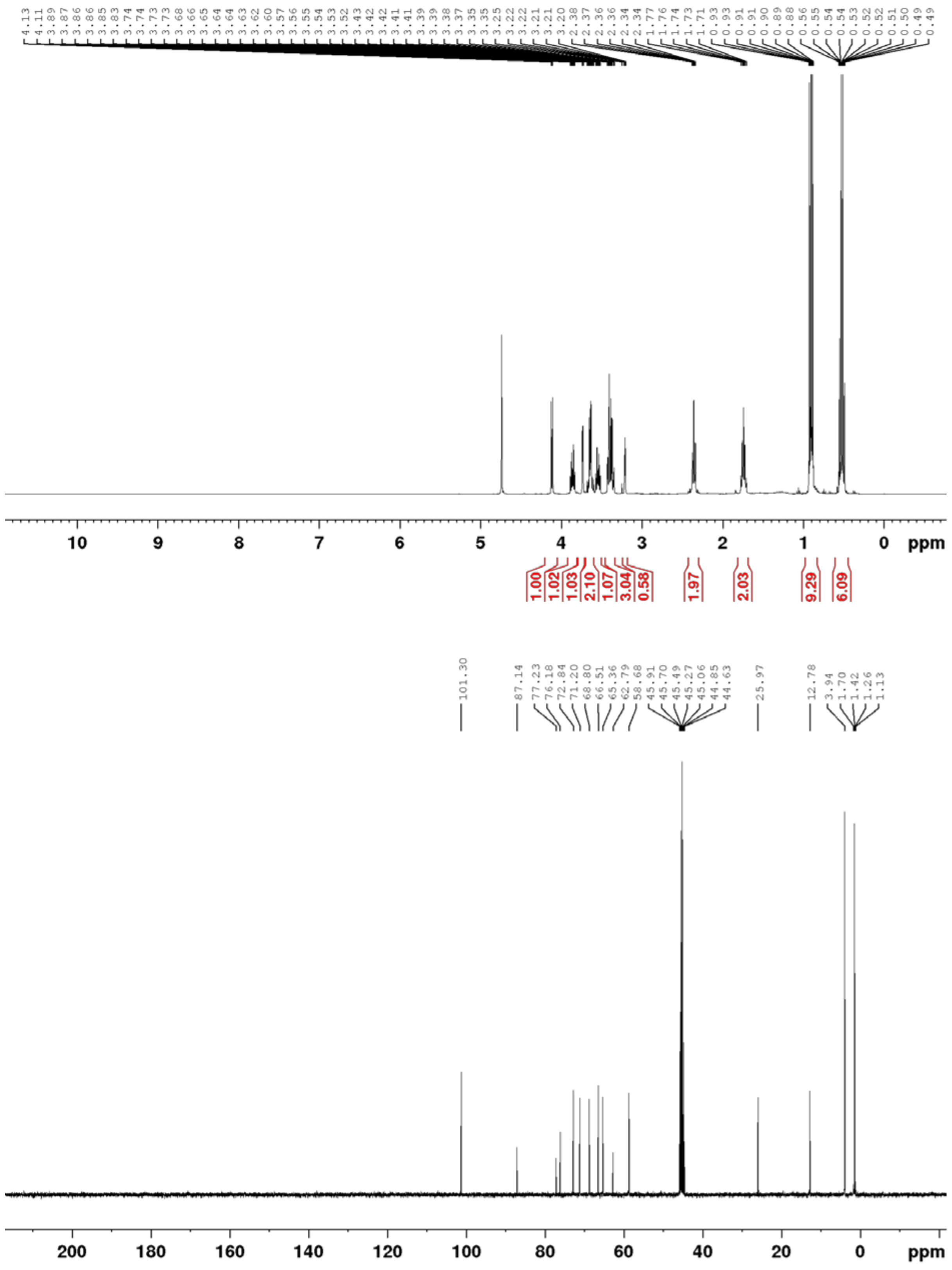

Hepta-4,6-diyn-yl-β-ᴅ-galactoside (2)

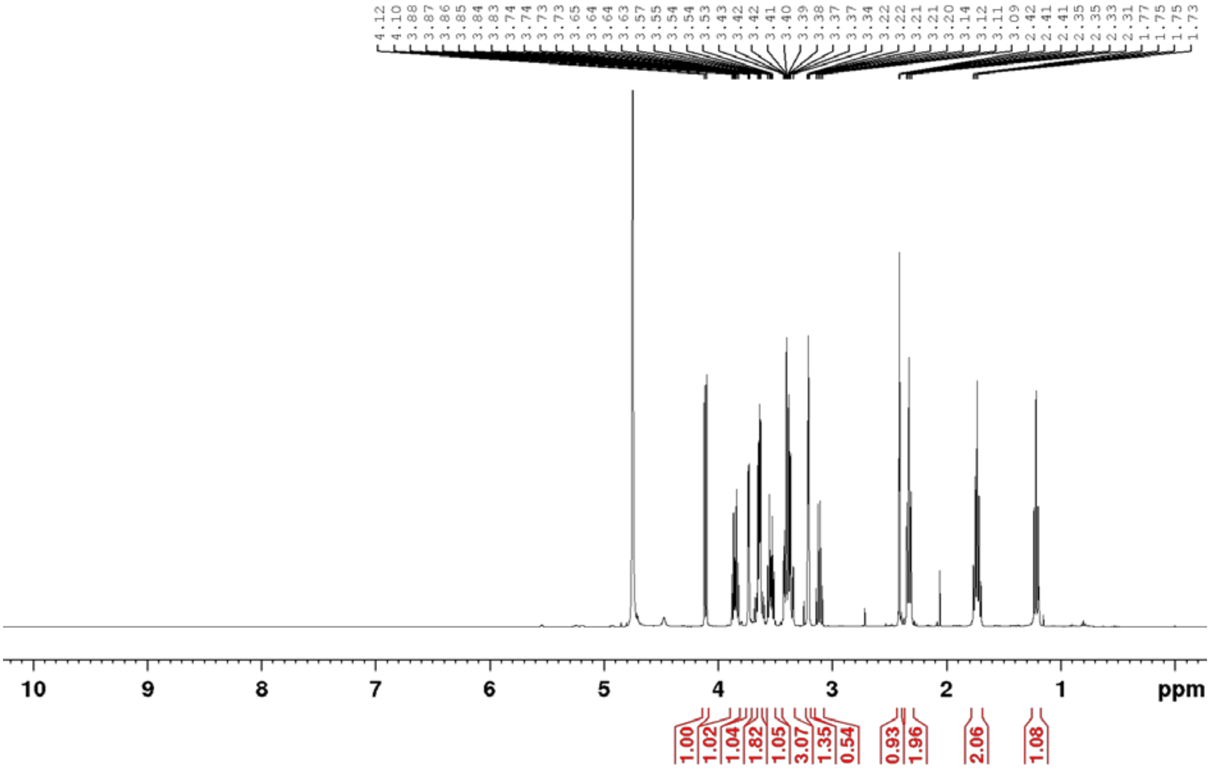

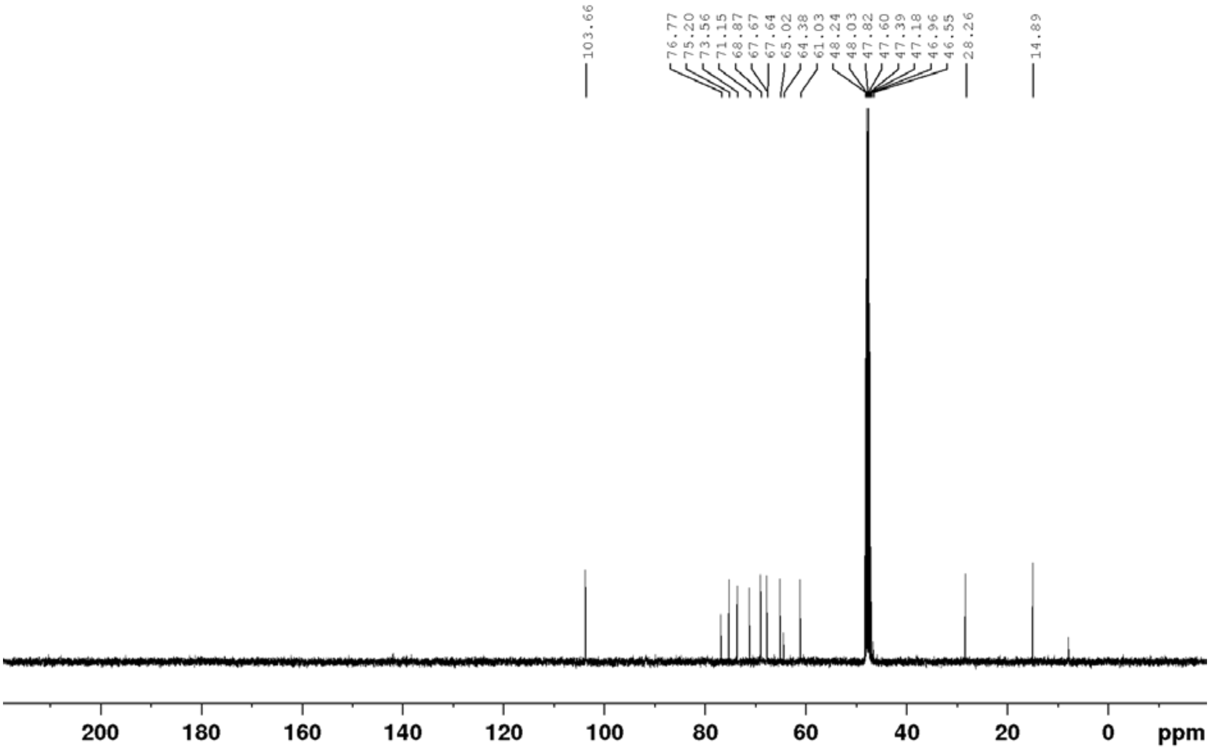

3-azido-7-hydroxycoumarin (3)

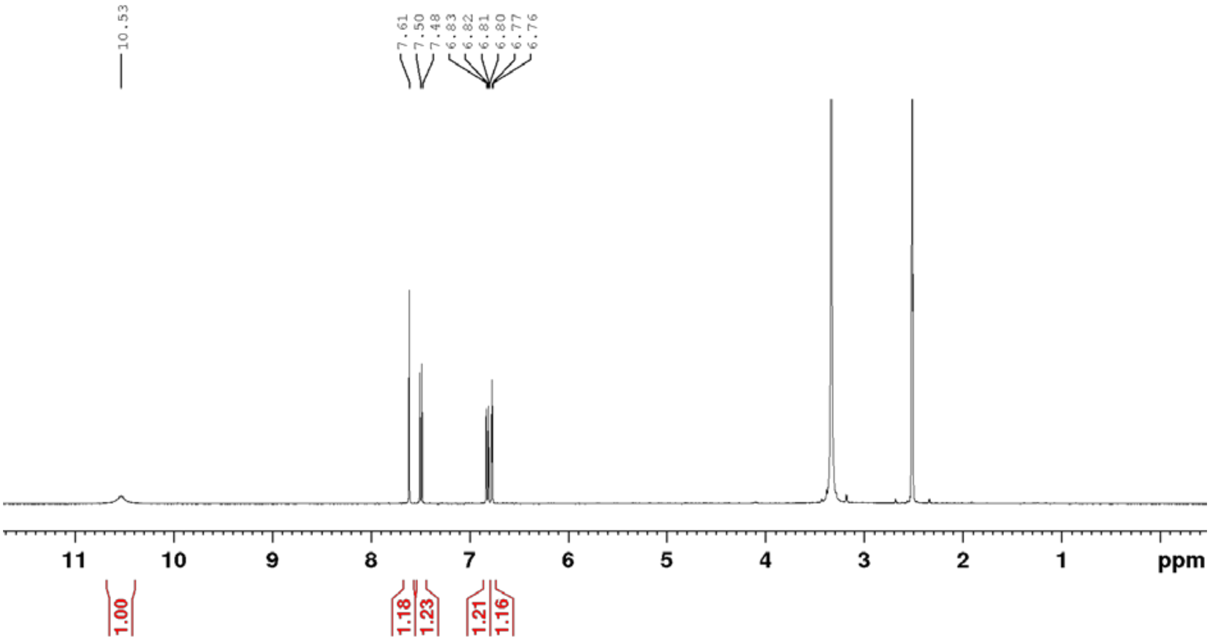


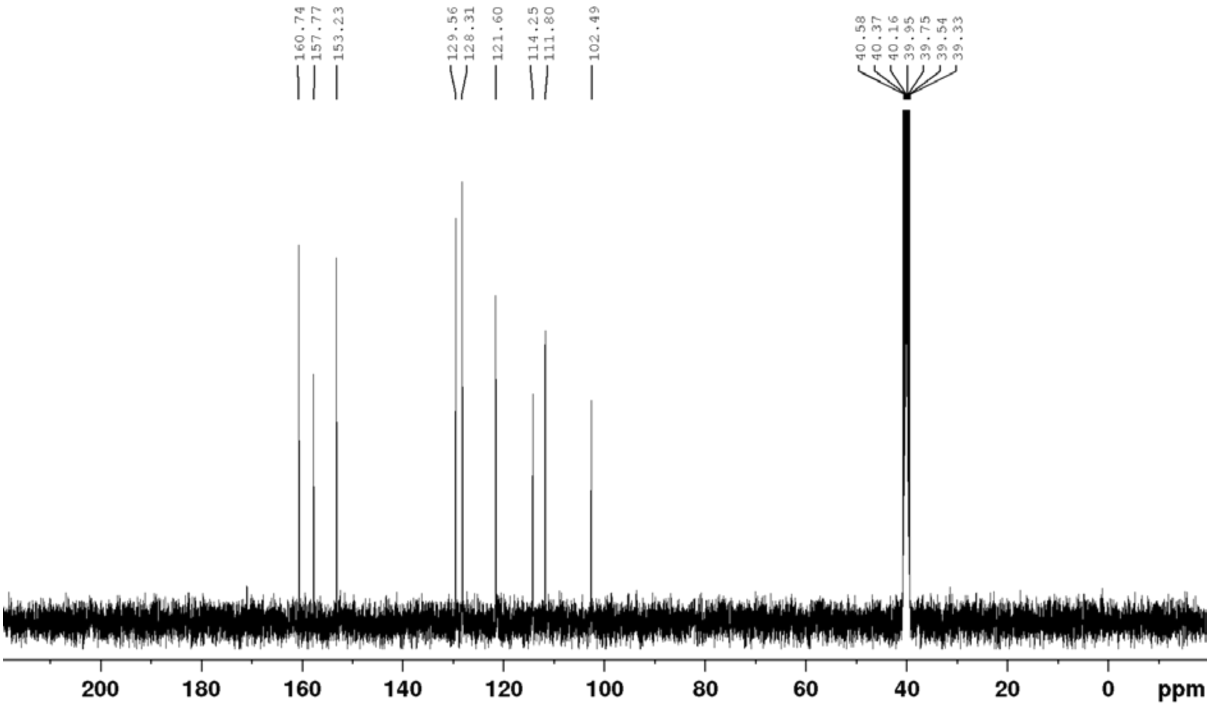

3-[1-(7-Hydroxy-coumarin)-1H-1,2,3-triazol-4-yl] propyl β-ᴅ-galactopyranoside (7)

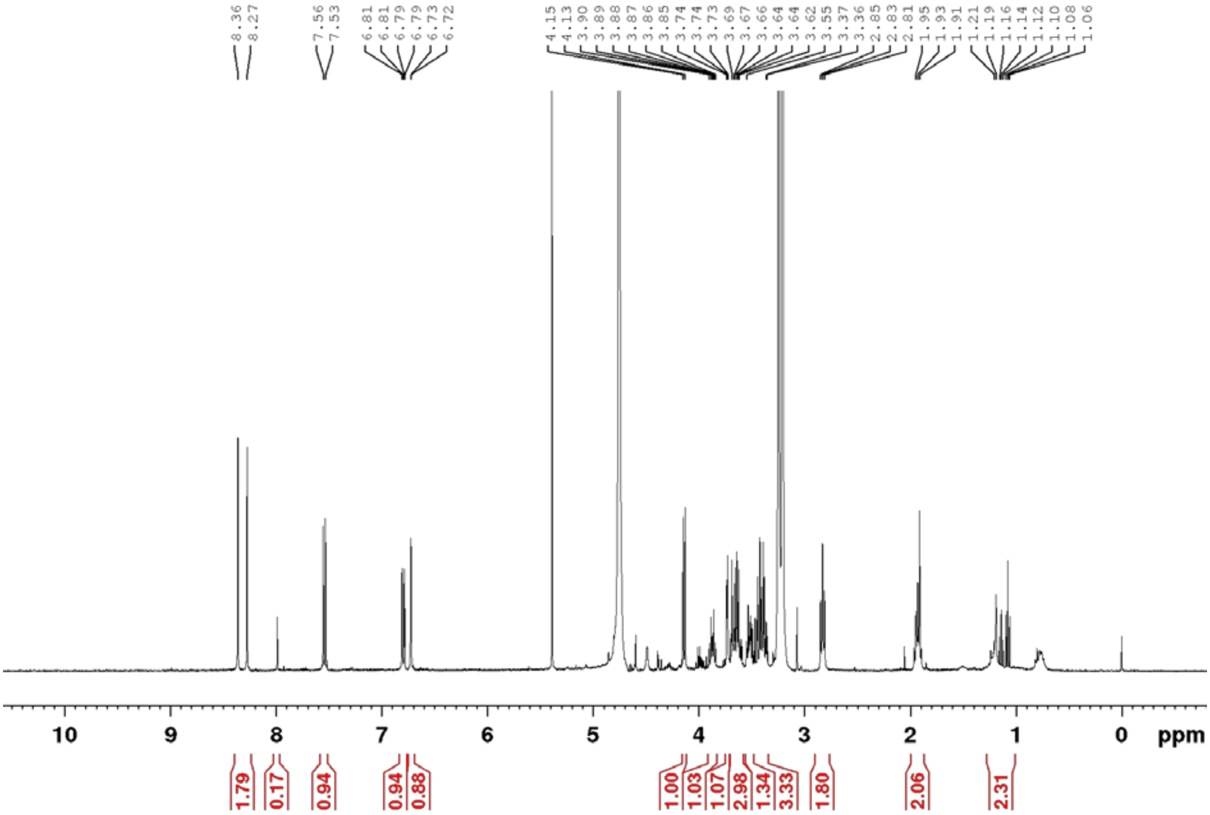


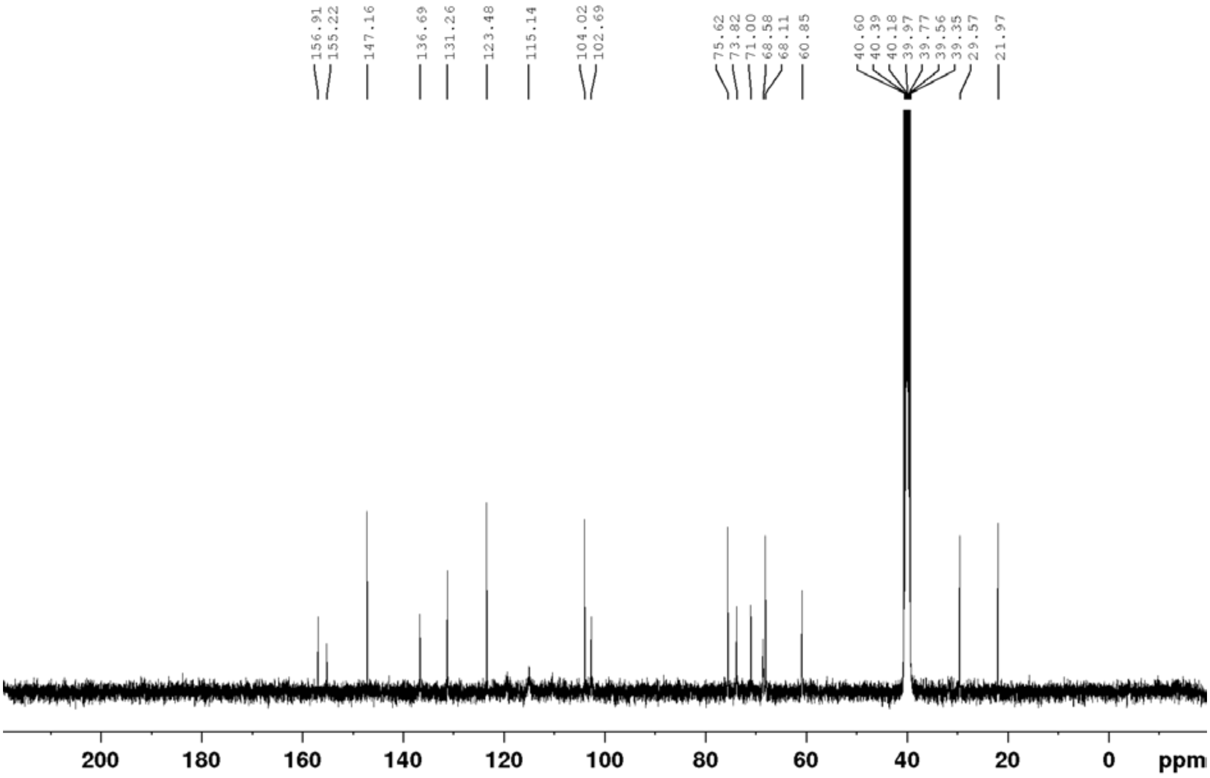


nb the 6'' carbon (115.4 ppm) was not clearly visible by $^{13}C$ NMR but was confirmed by HSQCed

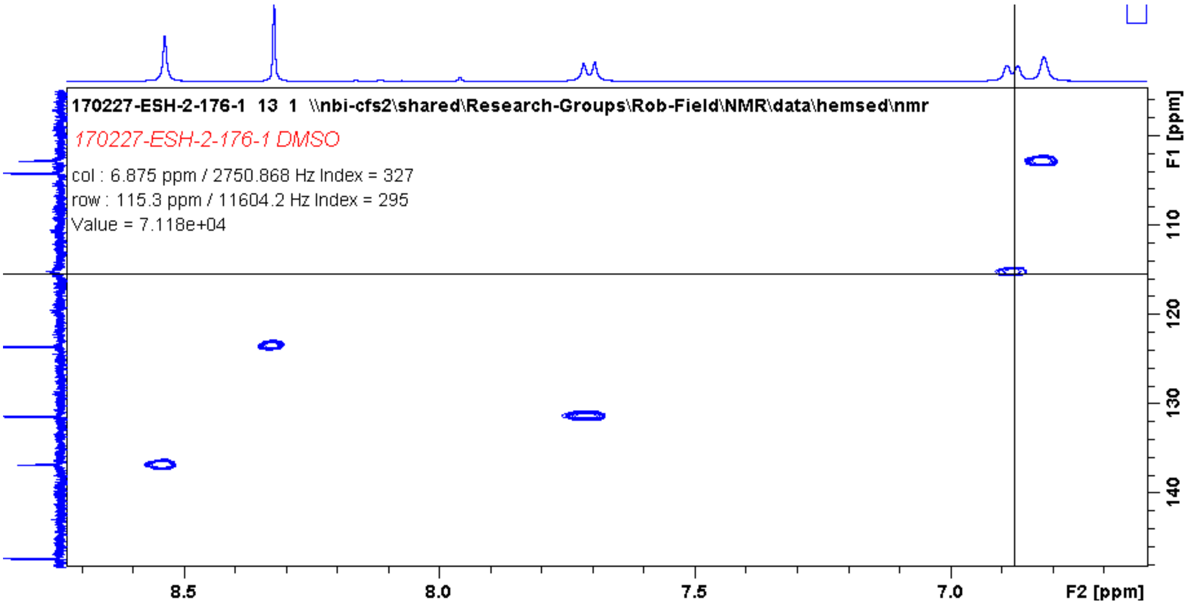


Zoomed HSQCed for compound **7** shows C6'' at 115.3 ppm

(for the purposes of this spectra, the $^{1}H$, $^{13}C$ and HSQCed were recorded in $d_6$-DMSO)

5-[1-(7-Hydroxy-coumarin)-1H-1,2,3-triazol-4-yl] pent-4-yn-1-yl β-ᴅ-galactopyranoside (8)

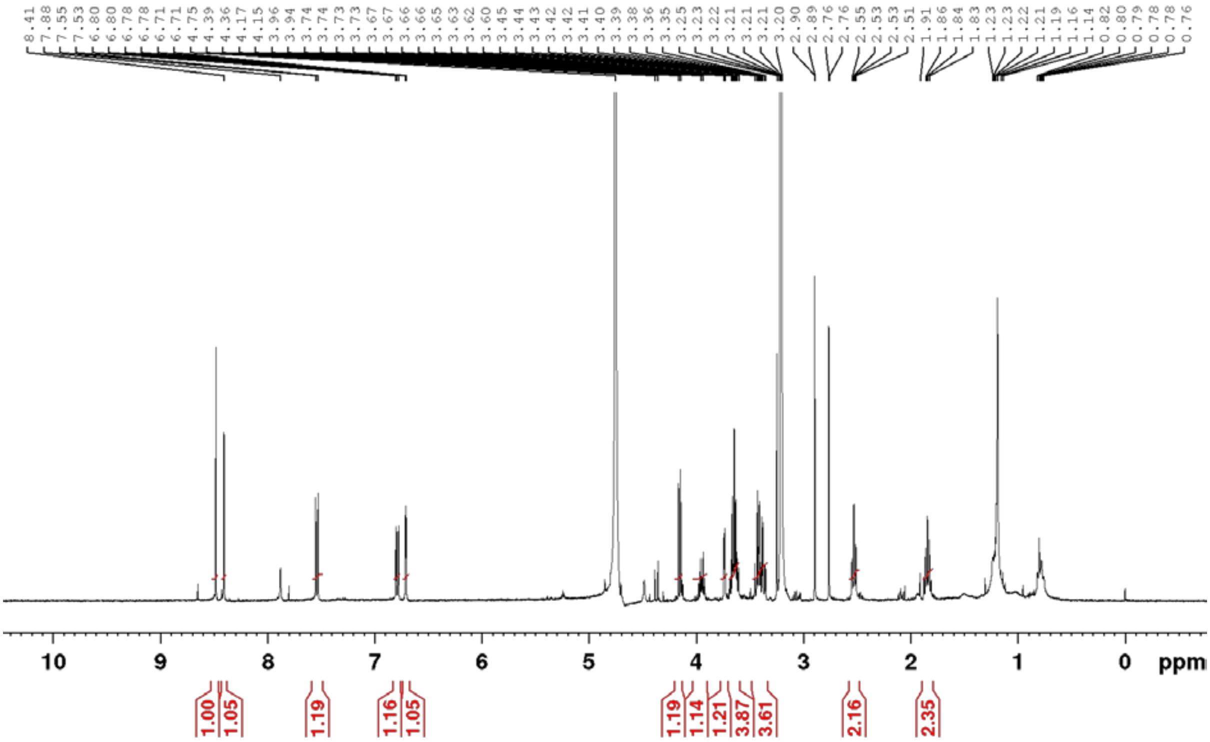


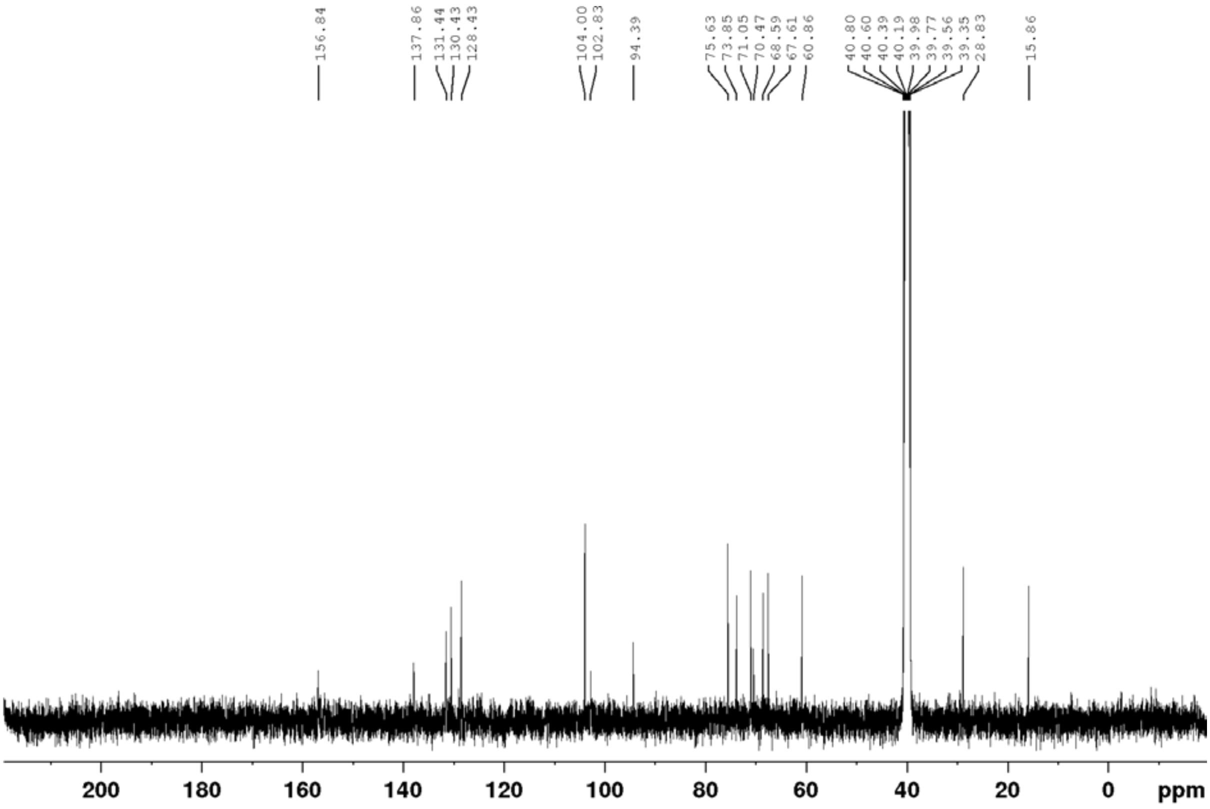


nb the 6’’ carbon was not visible by $^{13}$C NMR but was assigned by HSQCed

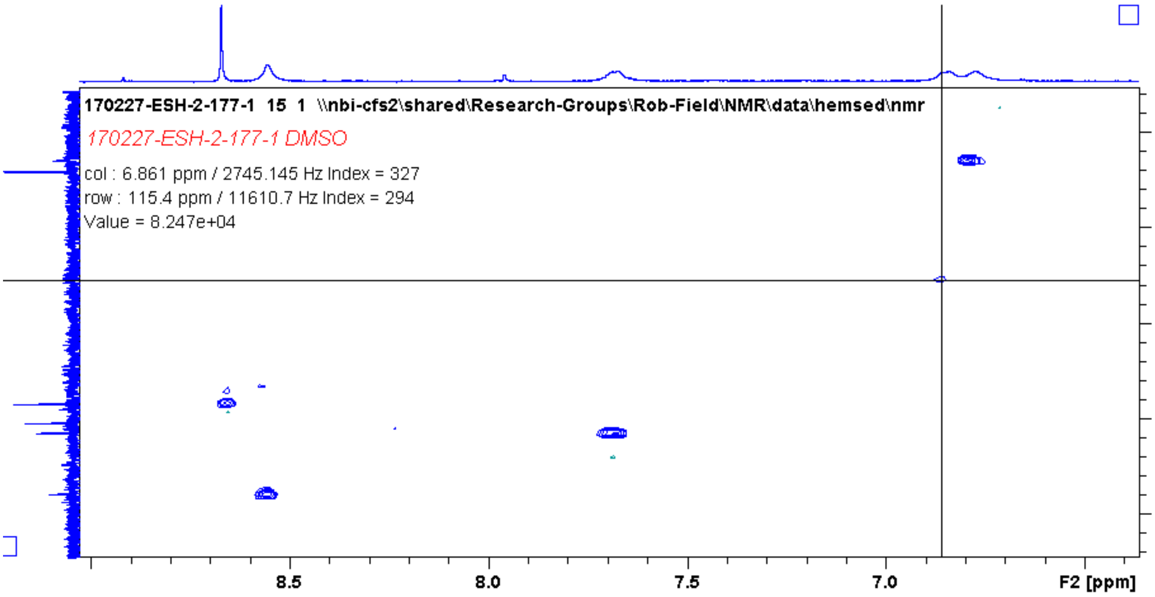


Zoomed HSQCed for compound **8** shows C6'' at 115.4 ppm

(for the purposes of this spectra, the $^{1}$H, $^{13}$C and HSQCed were recorded in $d_6$-DMSO)

# References:


1. M. Loos, C. Gerber, F. Corona, J. Hollender and H. Singer, *Anal. Chem.*, 2015, **87**, 5738-5744.
2. S. R. Manning and J. W. La Claire, *Anal. Biochem.,* 2013, **442**, 189-195.
3. B. A. Wagstaff, I. C. Vladu, J. E. Barclay, D. C. Schroeder, G. Malin and R. A. Field, *Viruses*, 2017, **9**, 40-51.
4. K. P. Wang, E. J. Cho, S. Y. Yun, J. Y. Rhee and D. Lee, *Tetrahedron*, 2013, **69**, 9105-9110.
5. F. Pertici and R. J. Pieters, *Chem. Commun.*, 2012, **48**, 4008-4010.
6. W. Y. Lu, X. W. Sun, C. Zhu, J. H. Xu and G. Q. Lin, *Tetrahedron*, 2010, **66**, 750-757.
7. K. Sivakumar, F. Xie, B. M. Cash, S. Long, H. N. Barnhill and Q. Wang, *Org. Lett.*, 2004, **6**, 4603-4606.
